# Inspired by Reinforced‐Concrete Structures: High‐Strength, Stable, Sustainable Reed‐Based Plastics

**DOI:** 10.1002/advs.202511564

**Published:** 2025-08-11

**Authors:** Yuhui Huang, Handong Li, Meng Li, Kui Li, Yawen Zhang, Zhen Zhang, Yingfeng Zuo, Yiqiang Wu

**Affiliations:** ^1^ State Key Laboratory of Utilization of Woody Oil Resource Central South University of Forestry and Technology Changsha Hunan 410004 P. R. China; ^2^ Huidong County Forestry Technology Promotion Station Huizhou Guangdong 516300 P. R. China; ^3^ College of Furniture and Art Design Central South University of Forestry and Technology Changsha Hunan 410004 P. R. China

**Keywords:** high strength, multiple bonding interface, reed‐based plastic, reinforced concrete structure, sustainability

## Abstract

Green and sustainable alternatives to petroleum‐based plastics can reduce environmental pollution and alleviate the resource crisis. Reed straw (Rs) is a rapidly growing, eco‐friendly, low‐cost, and natural fiber with great potential as a sustainable resource. However, the development of high‐performance plastic substitutes from Rs is challenging owing to its weak interfacial bonding. Herein, inspired by the structure of reinforced concrete, a top‐down strategy is adopted using a simple and rapid physical‐filling and directional‐assembly method in which the adhesive is evenly filled and adhered to the regular and continuous reed fiber skeleton. A reed‐based plastic with high strength, stability, fire resistance, and nontoxicity is developed using this approach via densification and rapid curing. The flexural strength, flexural modulus, and tensile strength of the material are 116 ± 8.05 MPa, 13.54 ± 0.67 GPa, and 81 ± 9.85 MPa, respectively. Moreover, the material demonstrated excellent dimensional stability during baking at 120 °C baking and 24 h of water immersion, and achieved a V‐0 rating in the UL‐94 test without producing toxic gases during pyrolysis. Benefiting from these properties, the developed reed‐based plastic demonstrates potential as a sustainable alternative to conventional petroleum‐derived plastics, with promising applications in home decor and structural construction.

## Graphical abstract

Inspired by the structure of reinforced concrete, which develops a composite with high‐strength, stability, fire resistance, and nontoxicity, by using the regular and continuous of reed fiber and thermoset tannin‐based resin. This composite demonstrates potential as a sustainable alternative to conventional petroleum‐derived plastics, with promising applications in home decor, structural construction, automotive interiors, and outdoor guardrails.

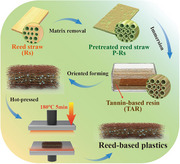

## 1 Introduction

Owing to their ease of processing, light weight, high strength, and low cost, plastics have brought great convenience to daily life.^[^ ^1^ ^]^ Plastic products have penetrated many industries, including home decoration, packaging, transportation, structural construction, and electronic information, and the total output of plastic products is estimated to exceed 11 billion tons by 2025.^[^ ^2^ ^]^ Plastics such as polyethylene (PE), polystyrene (PS), and polyvinyl chloride (PVA) are derived from nonrenewable petroleum and have a saturated carbon chain structure that requires hundreds of thousands of years to degrade naturally.^[^ ^3^, ^4^ ^]^ The use of plastics has led to their accumulation in nature, causing serious white pollution and irreversible damage to the global ecological environment,^[^ ^5^ ^]^ highlighting the need for renewable and biodegradable plastics.

Plant fibers are biodegradable and environmentally friendly biomass materials with abundant sources, rendering them promising substitutes for traditional plastics.^[^ ^6^ ^]^ In general, the plasticity and self‐adhesion of plant fibers are significantly lower than those of traditional plastics, thereby posing significant challenges in enhancing the mechanical strength and stability of bio‐based plastics. This limitation is commonly attributed to the large number of hydrophilic (hydroxyl) groups in the molecular chains of the fibers, which form weak noncovalent interfacial interactions during self‐adhesion.^[^ ^7^ ^]^ These bonds can be eroded by water molecules and weakened at high temperatures, significantly reducing the overall performance of the resulting products. Such defects can be resolved using various processes, including matrix extraction and purification,^[^ ^8^ ^]^ chemical functional‐group activation,^[^ ^9^ ^]^ coupling bridging,^[^ ^10^ ^]^ biological fermentation,^[^ ^11^ ^]^ and solvent melting‐casting.^[^ ^12^ ^]^ These methods directly alter the macrostructure, microstructure, and physicochemical properties of fibers to improve their plasticity and self‐adhesion. However, they also present significant challenges, including cumbersome processing steps and high manufacturing costs. Furthermore, biomass plastics are typically fabricated using a bottom‐up strategy in which fibers are disintegrated into fine‐scale units to maximize their interfacial contact surface area with chemical reagents, thereby improving modification efficiency.^[^ ^13^, ^14^ ^]^ However, this process increases the molecular spacing between binding units, and the product obtained after disorderly recombination often exhibits poor structural strength,^[^ ^15^, ^16^ ^]^ resulting in insignificant improvements in mechanical strength and stability, and ultimately restricting their potential applications. Consequently, developing a simple and sustainable fabrication process to produce high‐strength, robust, and durable bio‐based plastics holds significant importance.

Reed straw (Rs) is rich in high‐quality fibers, which are natural polymer materials.^[^ ^17^ ^]^ These fibers are strong and water‐resistant, thereby holding great potential in the field of bio‐based plastics. However, the waterproofness of the Rs is attributed to the presence of a hydrophobic siliceous layer covering the outer surfaces, which prevents self‐bonding between reed fibers, thereby constraining their development for bio‐based plastic applications.^[^ ^18^ ^]^ Although bottom‐up preparation strategies can partially remove this siliceous layer to increase fiber exposure and enhance self‐adhesion, it can also reduce the water stability of the reed fiber per unit volume.^[^ ^19^ ^]^ Researchers often add a bonding medium such as isocyanate, urea‐formaldehyde resin, soy protein, or tannin to enhance the interfacial adhesion and structural integrity between fibers.^[^ ^20^, ^21^, ^22^, ^23^, ^24^ ^]^ This method is fast, efficient, and cost‐effective. The resulting reed‐based products show considerable mechanical properties and stability. However, these products may release toxic gases such as toluene and formaldehyde during production or use, significantly restricting their practical applications. The other similar natural fiber composites have harsher fabrication conditions by comparison. For example, bamboo fibers require cumbersome preliminary processing as high‐temperature softening, flattening, and removing the outer skin.^[^ ^25^ ^]^ Hemp straws need complex chemical treatment to separate fiber bundles, a process that generates large amounts of wastewater.^[^ ^26^ ^]^ Moreover, the inherent longitudinal continuous fiber skeleton structure of Rs endows it with high toughness and strength.^[^ ^27^ ^]^ Bottom‐up strategies often overlook this critical aspect by disintegrating the fibers into fine particles that disrupt their intrinsic directional architecture, resulting in a significant decrease in the mechanical strength and stability of the resulting product. In contrast, the top‐down strategies have a more promising processing method. This method retains the original morphological and structural characteristics of the raw material, offering excellent structural strength for the composites.^[^ ^28^ ^]^ Meanwhile, top‐down strategies streamline the composite preparation process by eliminating steps like grinding and sieving, thereby reducing equipment costs for future industrial production. Consequently, a top‐down strategy is crucial for developing high‐strength reed‐based products that meet future industrial production requirements.

**Figure 1:**
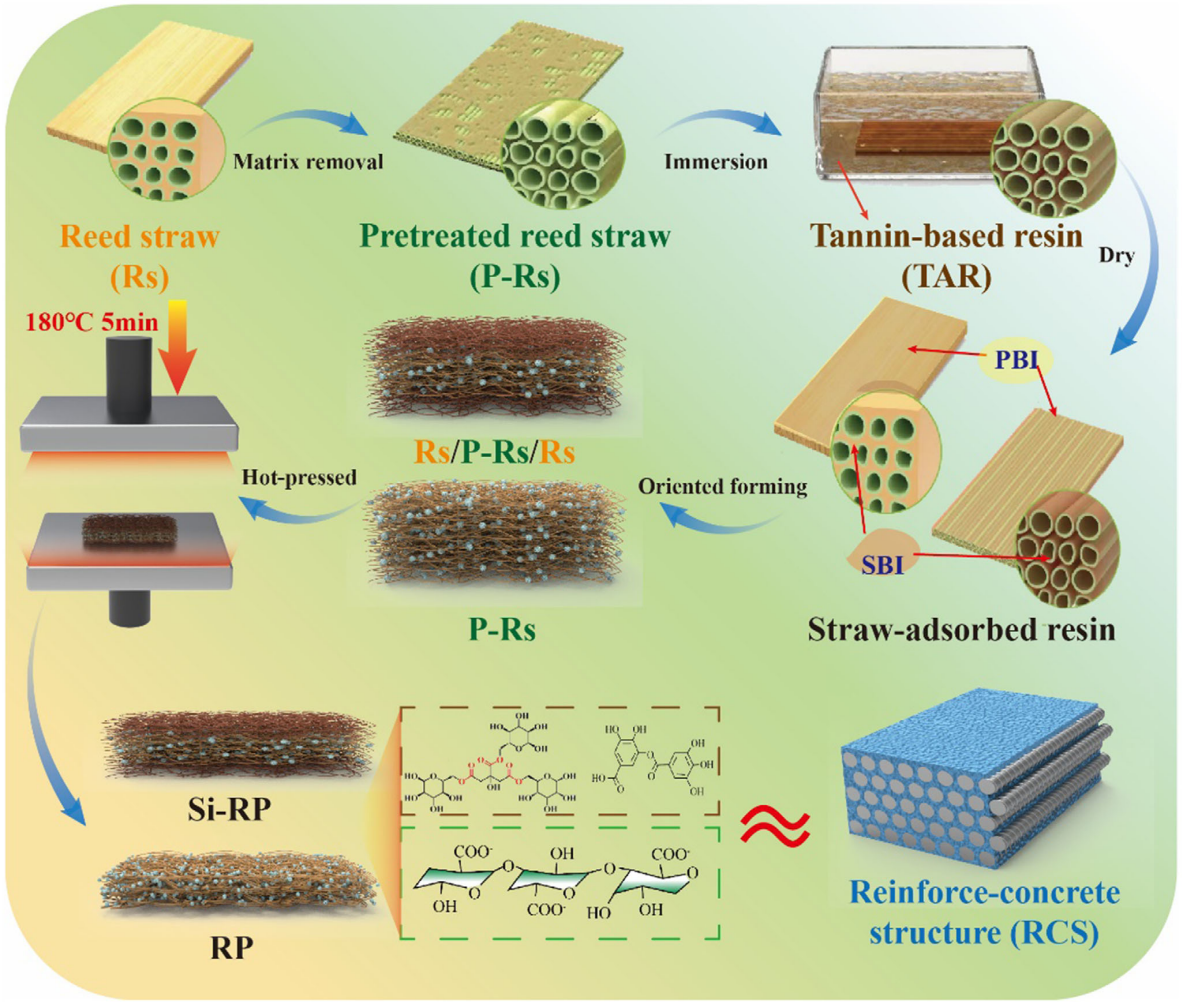
Schematic diagram of the preparation process of reed‐based plastics (Si‐RP and RP).

Large buildings generally use concrete to fill directional and ordered reinforcement frameworks to form a strong, tough, and durable reinforced‐concrete structure (RSC) after solidification.^[^ ^29^ ^]^ The reinforcement framework is ductile and tough, providing good tensile and bending stresses to the structure. The filling and adhesion of concrete to the reinforcement framework densify the structure and provide good stability. Consequently, inspired by the RCS and adopting a top‐down strategy, we report the use of the longitudinal continuous skeleton of reed fibers as a “reinforcement” to provide mechanical support. In this study, a self‐synthesized environmentally friendly tannin‐based resin (TAR) was used as an adhesion matrix that exhibits adhesion and hardening similar to those of concrete. Thus, the TAR is analogous to “concrete” and enhances the bonding force between reed fibers. “Plastic” is generally referred to as a synthetic polymer material, including thermosetting or thermoplastic systems.^[^ ^30^ ^]^ TAR is a thermosetting resin. Hence, we developed a reed‐based composite using reed fiber and TAR, and defined it as “reed‐based plastic”. This reed‐based plastic mimics an RCS through simple filling adhesion, directional stacking, and thermosetting molding (**Figure** **1**). The top‐down biomimetic design discussed in this paper eliminates the need to decompose continuous fibers into fine particles and then reassemble them, representing a simple and fast process for enhancing the structural strength of materials. Supported by this structure, the resulting reed‐based plastics exhibited excellent mechanical strength and stability, surpassing those of most traditional plastics. Reed‐based plastics are green, sturdy, and stable, and are strong candidates for replacing widely used petroleum‐based plastics in home decoration, packaging, transportation, and structural construction.

## 2 Results and Discussion

### 2.1 Morphology and Physicochemical Properties

**Figure 2:**
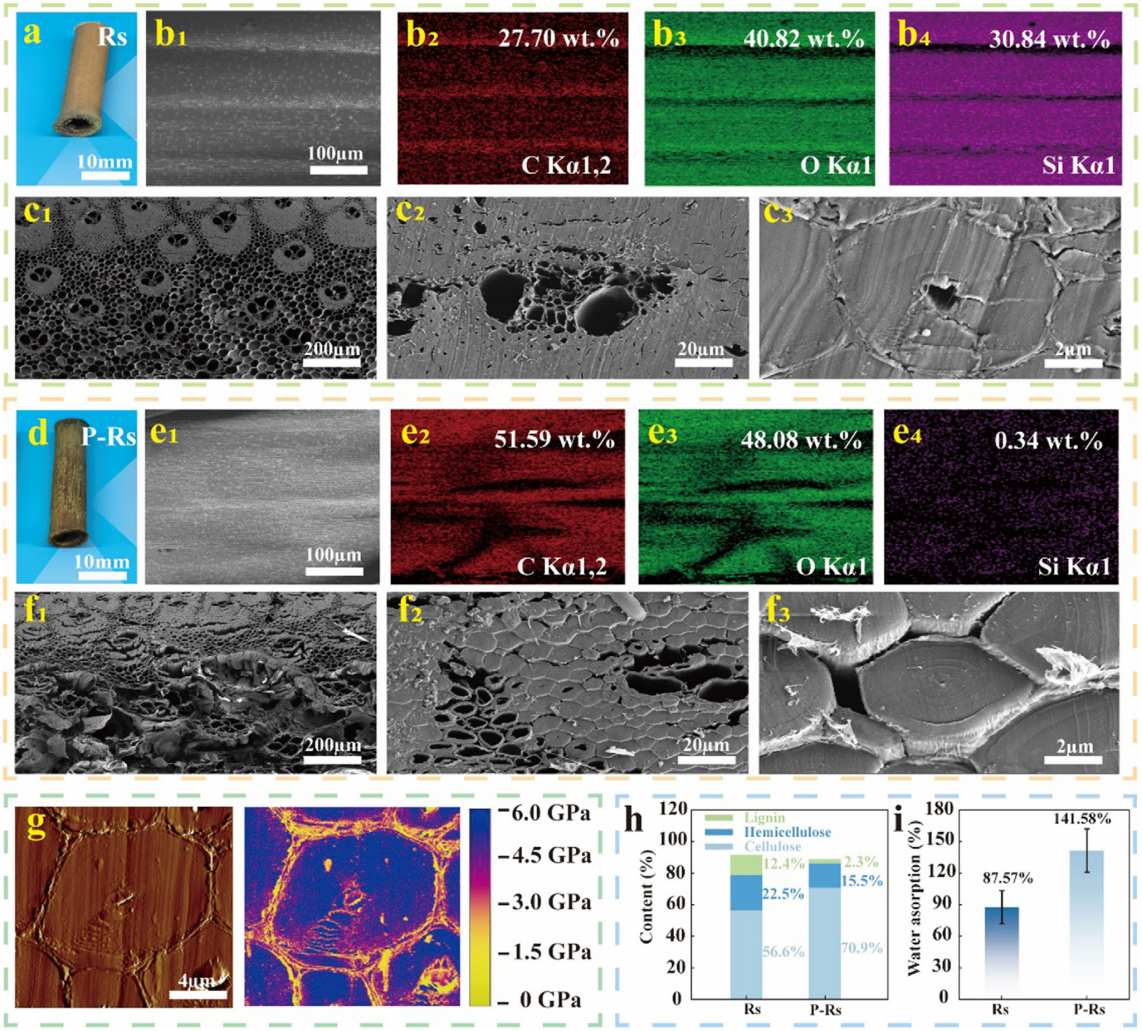
Morphology and physicochemical properties of Rs and P‐Rs. a, d) Photos of Rs and P‐Rs. b, e) Elemental maps of C, O, and Si in the outer epidermis of Rs and P‐Rs. c, f) SEM images of the fiber cross‐sections of Rs and P‐Rs. g) AFM images of the morphology (left) and DMT modulus (right) of the fiber cross‐section of Rs. h) Relative contents of cellulose, hemicellulose, and lignin in Rs and P‐Rs. i) WA rates of Rs and P‐Rs.

Scanning electron microscopy‐energy dispersive X‐ray spectroscopy (SEM‐EDS) was used to examine the microstructure of Rs. The outer layer of untreated Rs was covered with a regular and dense nonpolar siliceous material (**Figure** **2**a,b), which was hydrophobic and easily blocked the penetration of water (Figure S1, Supporting Information). After alkali treatment, the siliceous layer of the pretreated Rs (P‐Rs) was largely removed, exposing the internal fibers and improving their wettability (Figure 2d,e; Figure S1c, Supporting Information). This step provides favorable conditions for the water‐based adhesive medium to penetrate the internal structure of the reed fibers and form strong bonds. Reed is a natural fiber‐reinforced composite with high toughness owing to its gradient hierarchical structure composed of a vascular bundle and parenchymal tissues, which are dense on the outside and loose on the inside (Figure 2c). The vascular bundle is composed of dense rigid fibers that are mainly composed of cellulose, hemicellulose, and lignin.^[^ ^31^ ^]^ This structure provides the main mechanical support for Rs. However, the tissue structure of P‐Rs was severely damaged by alkali treatment, resulting in the appearance of numerous cracks between fiber cells, and its overall structure became loose (Figure 2f). This phenomenon was attributed to the fact that the intercellular layer of the fibers (yellow area) is a weak area (Figure 2g) that is mainly composed of pectin and easily soluble in alkaline solution.^[^ ^32^, ^33^ ^]^ However, the loose structure of P‐Rs provides high porosity and permeability pathways for the adhesive medium (Figure S2, Supporting Information). The alkaline solution also eroded the fiber cells through the intercellular layer, resulting in the partial removal of the matrix. Compared with Rs, the relative contents of lignin and hemicellulose in P‐Rs decreased from 12.4% and 22.5% to 2.3% and 15.5%, respectively. The relative lignin content also decreased significantly (Figure 2h). Consequently, the FTIR spectra of P‐Rs showed decreases in the intensities of lignin peaks at 1720 cm^−1^ (C═O stretching vibration), 1603 and 1510 cm^−1^ (C═C stretching vibration), and 1250 cm^−1^ (C─O stretching vibration of the syringyl unit) (Figure S3a, Supporting Information).^[^ ^34^, ^35^ ^]^ By contrast, its relative cellulose content increased, helping the liquid to penetrate evenly into the reed fibers, causing the water absorption (WA) rate of P‐Rs to reach 141.58% within 5 min (Figure 2i). In addition, the crystallinity of the cellulose improved (Figure S3b, Supporting Information), increasing the rigidity of the reed fibers. Prolonged high‐temperature alkaline treatment resulted in adverse effects, particularly cracks within the internal continuous reed fibers (Figure S4, Supporting Information). These cracks may act as fracture initiation points during tensile loading, thereby reducing the inherent strength of the fibers (Figure S5, Supporting Information).

**Figure 3:**
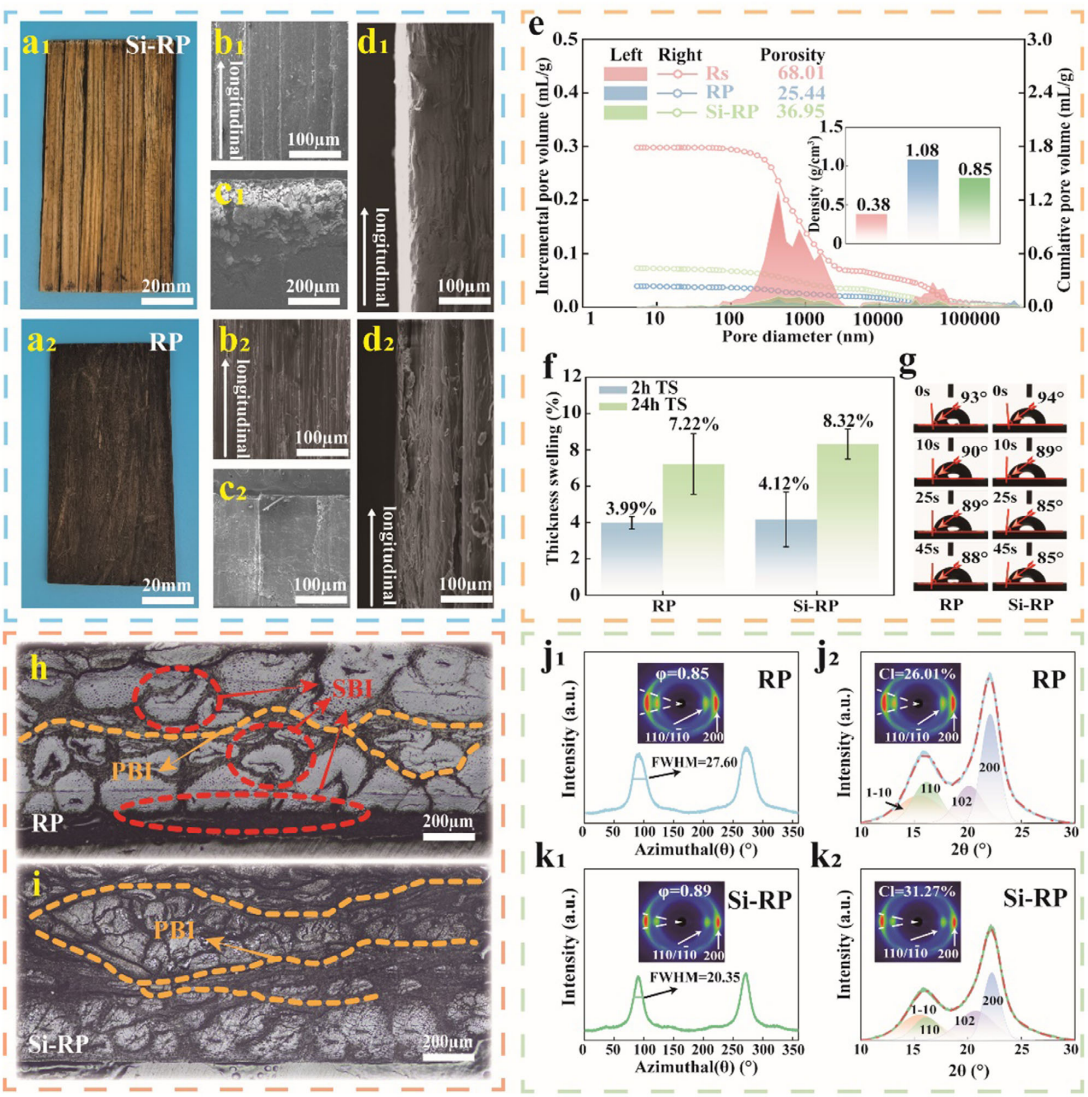
Morphology, structure, and physicochemical properties of Si RP and RP. a–d) Photographs and SEM images of Si‐RP and RP at different viewing angles. e) MIP data of Si‐RP and RP, including their incremental pore volume, cumulative pore volume, porosity, and density. f) TS rates of Si‐RP and RP after 2 and 24 h WA. g) Dynamic water contact angles of Si‐RP and RP. h,i) Ultra‐depth 3D image of the bonding interface between fibers in Si‐RP and RP. j,k) 2D‐WAXD images of Si‐RP and RP. The *φ* values of RP (j_1_) and Si‐RP (k_1_) were calculated from the azimuth integral curves of the (200) crystal planes. The *CI* values of RP (j_2_) and Si‐RP (k_2_) were calculated from the area integral curves of the (200, 110, 102, and 1‐10) crystal planes.

The RCS‐inspired reed‐based plastics were constructed using reed fibers (Rs and P‐Rs) as the reinforcement skeleton and TAR as the concrete layer through a series of processes (Figures 1 and **3**a). Here, the reed‐based plastics prepared using Rs and P‐Rs were labeled Si‐RP, whereas the reed‐based plastics prepared using only P‐Rs were labeled RP. The macrodensity of Si‐RP was significantly lower than that of RP (Figure 3b–d). Mercury intrusion porosimetry (MIP) showed that the air‐dry density and porosity of Si‐RP were 0.85 g cm^−3^ and 36.95%, respectively, while those of RP were 1.08 g cm^−3^ and 25.44%, respectively (Figure 3e). This finding is mainly attributed to the good permeability and WA of P‐Rs, which allowed aqueous TAR to penetrate deeply and adsorb onto both its internal and external parts. By contrast, the complete and organized structure of Rs impedes the deep penetration of TAR into its internal fiber cells within a short time. Thus, the formation of a primary bonding interface (PBI) between P‐Rs by TAR and a secondary bonding interface (SBI) between loose fiber cells was due to the penetration of TAR through the intercellular layer (Figure 3h). After directional assembly and hot‐pressing, the reed fibers and TAR were combined in a multiscale manner to obtain a high‐density RP (Figure S6, Supporting Information). However, the Rs on Si‐RP can only adsorb TAR through the fibers exposed on the inner surface and forms a PBI with the P‐Rs in the middle layer in the form of “anchors.” The fibers inside Rs fail to bind with TAR to form an SBI, thereby preventing the surface layer of Si‐RP from forming strong bonds through multiscale binding after densification (Figure 3i). Thus, its density was consistently lower that of RP (Figure S7, Supporting Information).

Densification reduces the spacing between cellulose molecules, which is conducive to strengthening the mechanical properties of fiber materials.^[^ ^36^ ^]^ We characterized the fiber arrangement and crystal structure of RP and Si‐RP using 2D‐wide‐angle X‐ray diffraction (2D‐WAXD), which revealed that both samples showed four bright spots on their equator, representing the overlapping (110) and (1‐10) crystal planes and (200) structure of anisotropic Iβ cellulose fibers (Figure 3j,k).^[^ ^37^ ^]^ The bright spots of RP were significantly larger than those of Si‐RP. The orientation index (*φ*) and crystallinity index (*CI*) values (0.91% and 31.27%, respectively) of the Si‐RP nanocellulose fibers were significantly higher than those of the RP fibers (0.85% and 26.01%, respectively), indicating that Si‐RP has a more highly ordered crystal structure. This phenomenon may be attributed to Rs functioning as the outer layer of Si‐RP, with its surface doped with densely ordered siliceous materials, thereby enhancing the orientation and crystallinity of the latter. This feature also provides mechanical strengthening to the material. We further verified the water stability of the reed‐based plastics based on material reinforcement. The 2 and 24 h thickness swelling (TS) rates of RP and Si‐RP were 3.99% and 7.22%, 4.12% and 8.32%, respectively (Figure 3f). RP showed good dimensional stability during long‐term immersion owing to its low porosity, which weakened erosion by water molecules (Figure 3g; Figure S10, Supporting Information). TAR solidified inside the RP forming substances that do not form hydrogen bonds with water molecules. These substances may contain C═O, C─O─C, and other groups (Figures S8 and S9, Supporting Information). Although the porosity of Si‐RP was high, the siliceous hydrophobic layer on the surface of Rs provided impermeability (Figures S11 and S12, Supporting Information),^[^ ^38^ ^]^ giving it good dimensional stability.

### 2.2 Mechanical Properties

**Figure 4:**
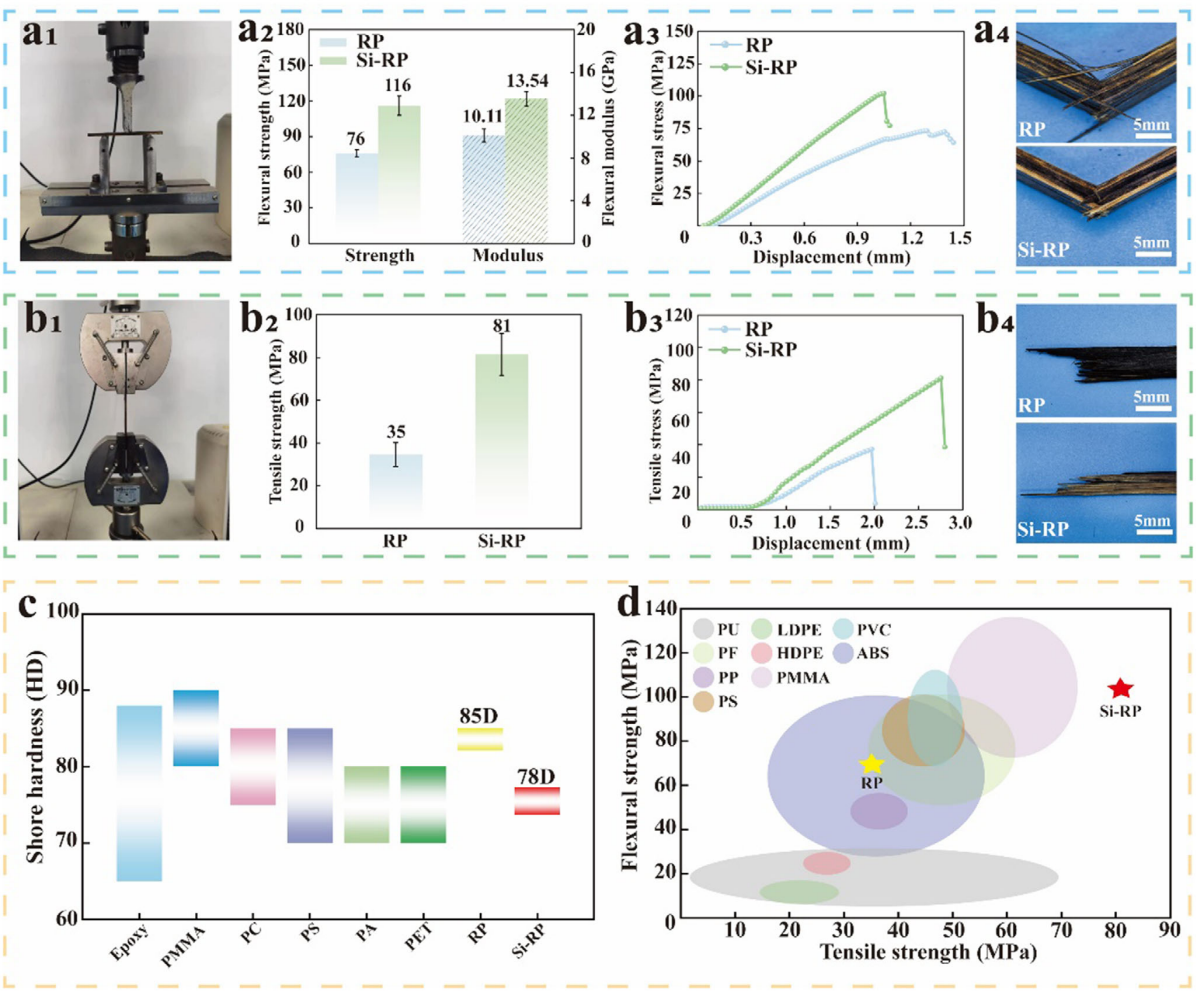
Mechanical properties and failure modes of Si‐RP and RP. a) Flexural properties (a_1_) of Si‐RP and RP, including their flexural strength and modulus (a_2_), flexural stress displacement curves (a_3_), and flexural failure mode (a_4_). b) Tensile properties of Si‐RP and RP (b_1_), including their tensile strength (b_2_), tensile stress displacement curve (b_3_), and tensile failure mode (b_4_). c,d) Comparison of the hardness and flexural‐tensile strength between Si‐RP, RP, and traditional plastics.

The flexural strength, flexural modulus, and tensile strength (**Figure** **4**) of Si‐RP in the dry state were 116 ± 8.05 MPa, 13.54 ± 0.67 GPa, and 81 ± 9.85 MPa, respectively (Figure 4a_2_–b_2_). These values were much higher than those of RP (76 ± 2.97 MPa, 10.11 ± 0.61 GPa, and 35 ± 5.59 MPa, respectively). The tensile strength of Si‐RP, which was 2.3 times that of RP (Table S2, Supporting Information), was particularly high. Under the same displacement conditions, Si‐RP displayed greater flexural‐tensile stress than RP (Figure 4a_3_‐b_3_). When subjected to flexural stress, the fibers on the RP fracture surface were interlaced and fractured, whereas those on the Si‐RP fracture surface remained smooth (Figure 4a_4_). This result can be attributed to the occurrence of continuous crack propagation on the surface of RP. The fiber bundles of RP were wrapped by the brittle TAR curing layer, leading to the occurrence of brittle fracture on the cured layer when subjected to flexural stress because it cannot bind the fiber bundles. The surface of Si‐RP was covered with ductile Rs, which significantly inhibited crack propagation. Therefore, when subjected to tensile stress, the fibers on the RP fracture surface were directly broken, and multiple single fibers were pulled out from the Si‐RP fracture surface (Figure 4b_4_). This phenomenon was attributed to the high crystallinity of P‐Rs (Figure S4b, Supporting Information), giving the fiber skeleton high rigidity.^[^ ^39^ ^]^ A brittle solidified layer of TAR covered P‐Rs, further increasing the brittleness of RP. However, the coverage of Rs the surface of Si‐RP increased its toughness. According to GB/T 4897‐2015, the mechanical properties of RP and Si‐RP exceeded the P4‐grade requirements (under dry conditions: flexural strength ≥ 20 MPa, flexural modulus ≥ 3.1 GPa). Meanwhile, the mechanical properties (under dry conditions) of Si‐RP are superior to those of other natural fiber composites (Tables S3 and S4, Supporting Information). In addition, after immersion in both cold (23 ± 2 °C) and hot (70 ± 2 °C) water according to GB/T 4897‐2015, the flexural strength of RP were 21 and 18 MPa, respectively, while those of Si‐RP were 97 and 38 MPa, respectively (Figures S13 and S14, Supporting Information), exceeding the P12‐grade requirements (flexural strength ≥ 13.2 MPa). Thus, the reed‐based plastics exhibited excellent wet‐aging resistance and hydrothermal aging stability.

The shore hardness values of RP and Si‐RP were measured to be 85D and 78D, respectively (Figure 4c; Figure S15, Supporting Information). In general, plastics with shore hardness values between 70D and 90D are classified as hard plastics, indicating that RP and Si‐RP are both hard plastics.^[^ ^40^ ^]^ This category includes polyethylene terephthalate (PET), polyamide (PA), polycarbonate (PC), polymethylmethacrylate (PMMA), and polystyrene (PS). The hardness of RP was similar to that of these materials, whereas that of Si‐RP was slightly lower. This finding is attributed to the reduced distance between cellulose molecules in P‐Rs following densification, rendering the RP structure more compact and enhancing its surface hardness. However, the organizational structure of Rs was unable to facilitate the deep penetration of TAR and its adhesion with the fibers, resulting in numerous pores remaining on the surface layer of the Si‐RP and, hence, low surface hardness. The flexural‐tensile strength of Si‐RP was equivalent to that of rigid plastics, such as PMMA, polyvinyl chloride (PVC), and phenol‐formaldehyde (PF) resins, and much higher than that of rigid plastics such as low‐density polyethylene (LDPE), high‐density polyethylene (HDPE), and polypropylene (PP). The strength of RP was equivalent to that of acrylonitrile butadiene styrene (ABS) and PP, and its bending strength was much higher than those of LDPE and HDPE. It is benefited from complete structure of Rs providing mechanical strength support for Si‐RP. However, the P‐Rs in RP have been severely damaged by the alkaline solution, resulting in their flexural‐tensile strength is equivalent to that of ABS and PP, and the flexural‐tensile strength is much higher than that of LDPE and HDPE. Overall evaluation shows that, compared to traditional plastics, RP and Si‐RP have a significant advantage in mechanical properties. Among them, hard plastics such as PS, PF, and PVC have a wide range of applications, and reed‐based plastics are expected to potentially replace them.

### 2.3 Nanomechanical Mechanism via Molecular Dynamics Simulation

**Figure 5:**
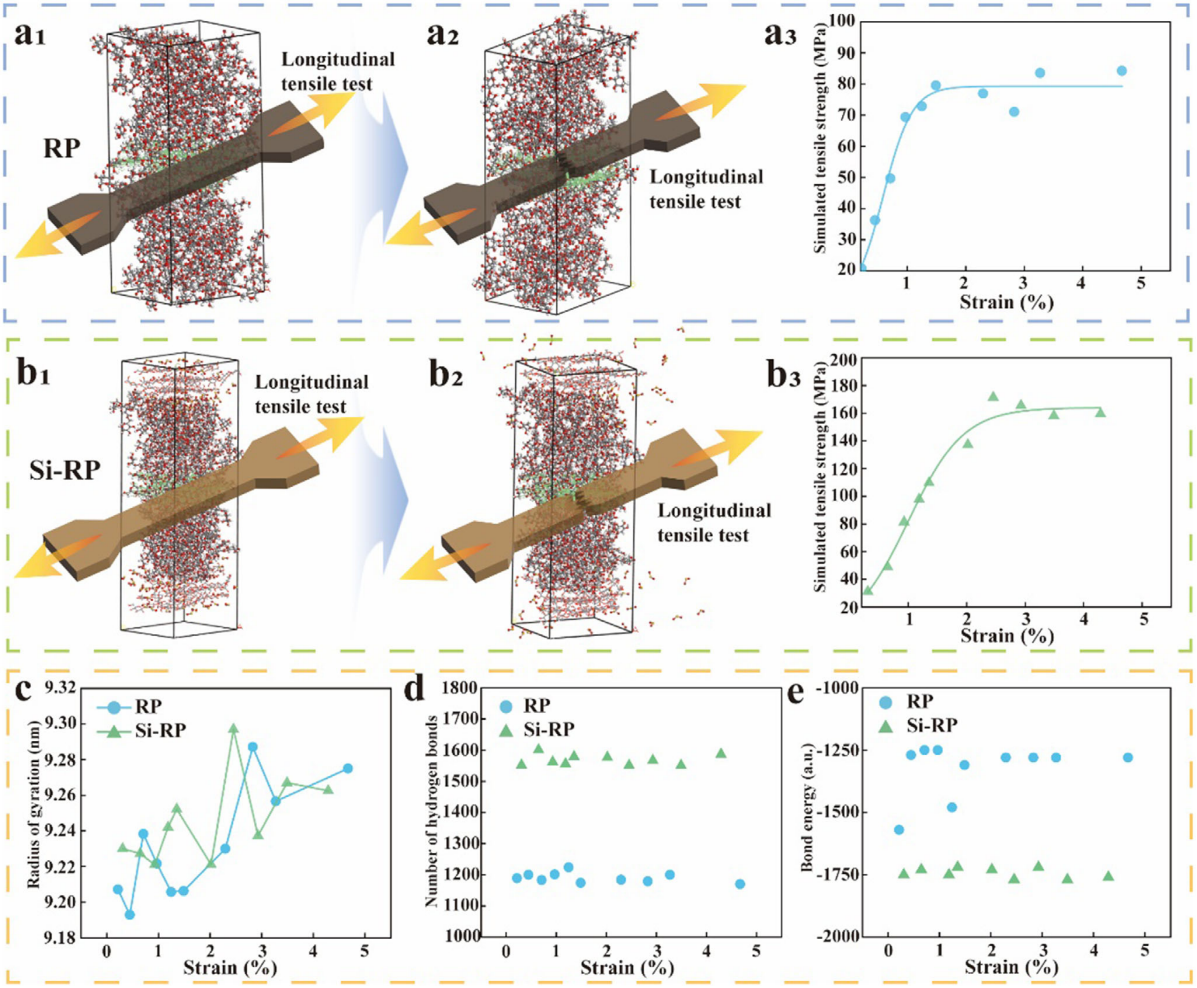
MD simulation of the tensile properties of Si‐RP and RP. a) Tensile stress‐strain curve (a_3_) of the RP model during tensile fracture (a_1_, a_2_). b) Tensile stress‐strain curve (b_3_) of the Si‐RP model during tensile fracture (b_1_, b_2_). c) Radius of gyration of the RP and Si‐RP model systems during tensile fracture. d) Number of hydrogen bonds in the RP and Si‐RP models. e) Bonding energies of the RP and Si‐RP models.

To reveal the molecular stretching mechanisms of RP and Si‐RP, we performed molecular dynamics (MD) simulations to determine the tensile strengths of the two systems using Large‐scale Atomic/Molecular Massively Parallel Simulator (LAMMPS) molecular simulation software. In the RP system, TAR was introduced into the oriented cellulose type *II* crystal framework (labeled as *II*), and a three‐layer model of TAR‐*II*‐TAR was established. In the Si‐RP system, the outer layer was the oriented cellulose *Iβ*@SiO_2_ mixed system (labeled as *Iβ*@Si) and the inner layer was the oriented *II* skeleton. TAR was introduced to construct a five‐layered model of *Iβ*@Si‐TAR‐*II*‐TAR‐*Iβ*@Si. Force‐field calculations showed that the maximum tensile strength in the fiber direction was 84.27 MPa for RP and 171.36 MPa for Si‐RP (**Figure** **5**a,b), which agreed with the trend of the experimental results. This result indicates that the RCS formed by the five‐layer model significantly enhances the tensile properties of the material. This model benefited from the excellent extensibility provided by the surface fibers (Rs) of Si‐RP, which enhanced its tensile properties. However, the experimental tensile strengths of RP and Si‐RP were lower than those obtained from the MD analysis, possibly because the MD analysis was conducted under uniform and defect‐free conditions.

The chemical compositions of different parts of Rs as well as the variations in the tensile properties of the reed‐based plastics prepared from them were further analyzed. The results demonstrated that the upper Rs section (U/Rs, the middle‐upper 1.5 m section) had a higher cellulose content and tensile strength than the basal Rs section (B/Rs, the middle‐lower 1.5 m section) (Figure S16, Supporting Information). However, after alkaline treatment, no significant differences in the components and tensile properties were found between U/P‐Rs and B/P‐Rs (Figure S16, Supporting Information). When these different straw parts were separately used to prepare RP and Si‐RP, their tensile strengths showed no significant differences within a 5% error (Figure S17, Supporting Information). This finding is attributed to the fact that long‐term high‐temperature alkaline treatment removes most of the lignin and hemicellulose from the reed fibers, thereby increasing the cellulose content and homogenizing it. Therefore, the homogeneity of the material had a relatively small impact on the tensile properties of the reed‐based plastics. However, during extended high‐temperature alkaline treatment, the reed fibers developed numerous microcracks (Figure S4, Supporting Information). These cracks served as initiation sites for fiber fracture during tensile loading, causing the material to fail through crack propagation and affecting the final experimental results.^[^ ^41^ ^]^ This deficiency is the primary factor contributing to the discrepancy between the experimental and MD data. The mismatch in the experimental scale between the experiment and MD simulations may also exacerbate these issues, ultimately causing the experimental and MD data to differ by 2–3 times.^[^ ^42^, ^43^ ^]^

To further reveal the nanoscale structural differences between RP and Si‐RP, the rotational radii of the two systems were calculated using MD simulations. During the tensile process, the radius of gyration of RP and Si‐RP increased with increasing tensile strain (Figure 5c) because the tensile stress transformed the cellulose molecular chains from a dense conformation to an extended conformation. This transformation was achieved by increasing the average distance between atoms in response to an external stress, increasing the radius of rotation.^[^ ^44^ ^]^ The observed trend revealed that Si‐RP has better extensibility than RP, consistent with the experimental results and indicating that the reinforced‐concrete microstructure assembled by *Iβ*@Si‐TAR‐*II*‐TAR‐*Iβ*@Si may be an effective method for preparing high‐strength reed‐based plastics. Compared with RP (≈1200 hydrogen bonds), Si‐RP (≈1550 hydrogen bonds) had significantly more hydrogen bonds, indicating the occurrence of significant interfacial interactions between the molecules in the Si‐RP system, as also evidenced by the molecular bonding energies obtained (Figure 5d,e; Figure S9 and Table S1, Supporting Information).^[^ ^45^ ^]^ Hence, the MD simulations indicated that the RCS formed by the five‐layer *Iβ*@Si‐TAR‐*II*‐TAR‐*Iβ*@Si model enhanced the mechanical properties of the reed‐based plastics.

### 2.4 Thermal Properties

**Figure 6:**
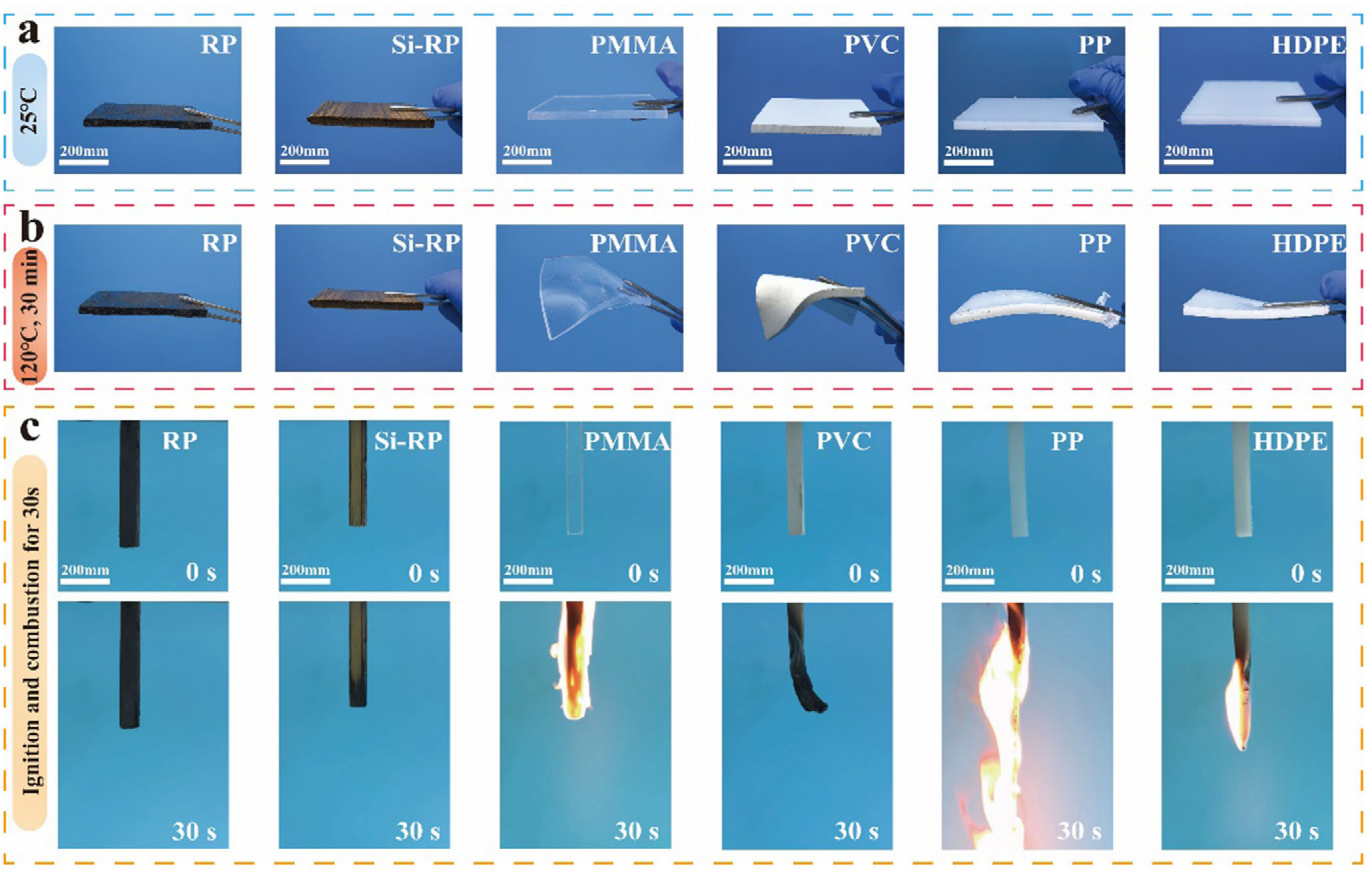
Comparison of the thermal dimensional stability and combustion performance between reed‐based plastics and traditional plastics. a,b) Photographs of the thermal deformation of RP, Si‐RP, PMMA, PVC, PP, and HDPE before and after heating. c) Photograph of the UL‐94 testing of RP, Si‐RP, PMMA, PVC, PP, and HDPE.

Traditional plastics easily undergo flexural deformation in high‐temperature environments, affecting their utility and appearance. Consequently, we compared the thermal deformation properties of the reed‐based plastics with those of traditional plastics subjected to high‐temperature treatment. When the reed‐based plastics (RP and Si‐RP) were baked for 30 min at 120 °C, they exhibited excellent thermal dimensional stability, whereas other traditional plastics, especially PMMA and PVC, underwent bending deformation (**Figure** **6**a,b). This result was attributed to the lower softening temperature of traditional plastics compared with that of fiber materials. At even higher temperatures, traditional plastics melt or burn, resulting in potential fire risks. The combustion performance of the reed‐based and traditional plastics was further compared using vertical combustion tests. After burning for 10 s in an open fire, the traditional plastics burned intensely and exhibited dripping combustion (Figure 6c; Figure S18, Supporting Information). By contrast, the reed‐based plastics were extinguished immediately after removal from the flame, indicating that their flame‐retarding properties are at the V‐0 level (Chinese National Standard GB/T 2408‐2021). This result was attributed to the presence of a biomass flame retardant (tannic acid, TA) in the reed‐based plastics, which quickly forms a carbonization layer on the surface of the reed fibers that isolates oxygen when exposed to open fire (Figure S19, Supporting Information), thus preventing the material from continuing to burn.^[^ ^46^ ^]^ To further assess the combustion performance of reed‐based plastics, cone calorimetry (CONE, which was set to a thermal radiation intensity of 50 kW/m^2^) was used for its characterization (Figure S20, Supporting Information), simulating real‐fire conditions. The reed‐based plastics have a time to ignition (TTI) of 37 s, total heat release (THR) of 20 MJ m^−2^, and total smoke release (TSR) of 63 m^2^ m^−2^ (Table S5, Supporting Information). Compared with petroleum‐based plastics, this finding indicates that reed‐based plastics are relatively flame‐retardant, thereby increasing the escape time for people before the fire spreads. Furthermore, it generated less smoke during combustion. This result was attributed to the formation of a char layer on the surface of reed‐based plastics during combustion. This layer acted as a barrier, preventing heat and oxygen exchange between the fibers and the external environment, and restricting the release of volatile compounds. Consequently, this mechanism contributes significantly to the observed smoke suppression effect.^[^ ^47^ ^]^ This smoke suppression plays an essential role in fire scenarios by aiding personnel evacuation and cutting inhalation exposure to smoke particulate matter. As a natural fiber‐reinforced composite, reed‐based plastics show remarkable flame‐retardant advantages over similar composites (Table S6, Supporting Information). Thus, reed‐based plastics are highly promising for use in home decoration, structural construction, and automotive interiors.

**Figure 7:**
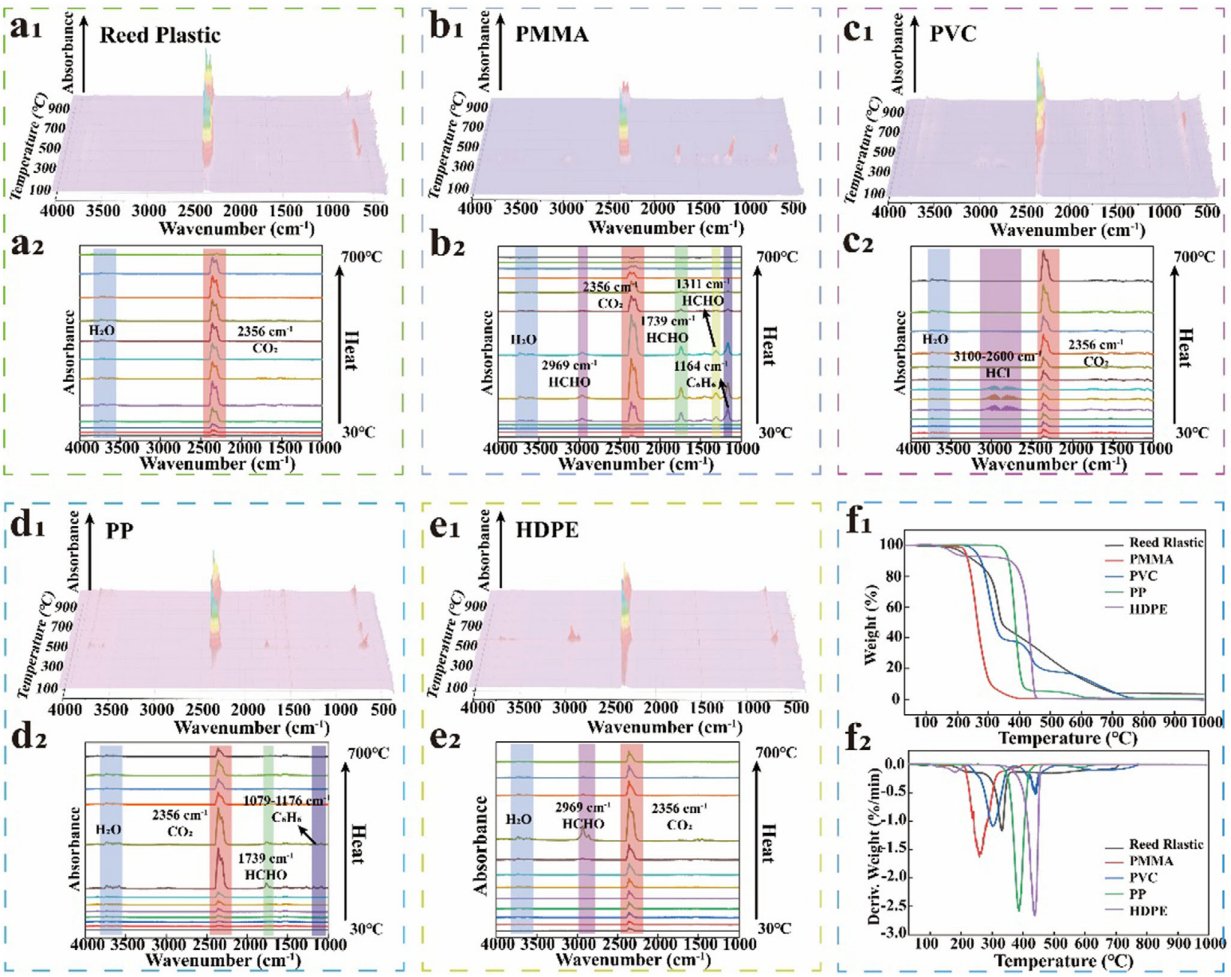
Comparison of pyrolysis products and thermal stability between the reed‐based plastics and traditional plastics. a–e) 3D‐FTIR spectra (a_1_‐e_1_) of the volatile products of the reed‐based plastics with PMMA, PVC, PP, and HDPE, and FTIR spectra (a_2_‐e_2_) of the pyrolysis products at different temperatures. f) Mass loss change (f_1_) and mass change rate (f_2_) of the reed‐based plastics and PMMA, PVC, PP, and HDPE.

Traditional plastics derived from petroleum may contain elements such as Cl, N, and F, rendering them susceptible to harmful gas emissions at high temperatures or during combustion.^[^ ^48^ ^]^ Hence, we characterized the pyrolysis products of the reed‐based and traditional plastics by Thermogravimetry‐Fourier transform infrared spectroscopy (TG‐IR). During pyrolysis, the PMMA and PP molecules underwent a series of complex reactions and produced volatile aromatic hydrocarbon compounds (**Figure** **7**b–d).^[^ ^49^ ^]^ The Cl atoms in the PVC molecules released a large amount of HCl gas through dehydrochlorination (Figure 7c).^[^ ^50^ ^]^ Formaldehyde is often used as a plasticizer to prepare traditional plastics^[^ ^51^ ^]^ leading to the release of formaldehyde gas during the pyrolysis of traditional plastics. Formaldehyde may also be freed during normal use (Figure 7b–e). By contrast, the reed‐based plastics only generated CO_2_ gas during pyrolysis (Figure 7a), indicating that they do not include toxic elements. After pyrolysis, the residual carbon content of the reed‐based plastics was 3.53%, whereas that of the traditional plastics was 0%, indicating thermal stability (Figure 7f). The thermal stability of reed‐based plastics is comparable to that of other natural fiber composites with fire retardants (Table S5, Supporting Information). Consequently, reed‐based plastics are green and environmentally‐friendly materials that can potentially replace traditional plastics, thereby facilitating sustainable development.

## 3 Conclusion

Inspired by the structure of reinforced concrete, we propose a simple and rapid molding method for fabricating reed‐based plastics. A multiscale bonding interface between TAR and reed fibers was achieved by using a top‐down strategy, including rapid and efficient filling‐adhesion and directional‐stacking methods. A high‐pressure rapid thermosetting process was used to form materials with microstructures similar to those of reinforced concrete. The reed‐based plastics exhibited high strength, good stability, fire resistance, and non‐toxicity, and their maximum flexural strength, flexural modulus, and tensile strength reached 116 ± 8.05 MPa, 13.54 ± 0.67 GPa, and 81 ± 9.85 MPa, respectively. The reed‐based plastics maintained good dimensional stability when baked for 30 min at 120 °C and soaked in water for 24 h. The combustion level of the materials reached the highest (V‐0) level, and no toxic gases were generated during high‐temperature pyrolysis. This material is expected to replace traditional plastics in home decorations, structural buildings, automotive interiors, and outdoor guardrails.

## 4 Experimental Section

### Materials

Rs (Arundo donax L.) with a length of 2.6–3.0 m was collected from Dongting Lake, Hunan, China. Citric acid (CA, C_6_H_8_O_7_·H_2_O), glucose (G, C_6_H_12_O_6_), and NaOH (≥ 99.5%) were obtained from Sinopharm Chemical Reagent Co., Ltd. TA (C_76_H_52_O_46_) was purchased from Xilong Science Co., Ltd.

### Pretreatment of Rs

The sturdy part of Rs was divided into two along its longitudinal direction sawed into 100 mm sections, and immersed in a 1.0 wt.% NaOH solution to remove part of the matrix. The process was carried out in a water bath at 85 °C for 3 h. Subsequently, the pretreated Rs was washed with deionized water until neutral and dried in an oven at 60 °C to a moisture content of ≈2–3%. The final pretreated Rs sample was labeled as P‐Rs.

### Preparation of TAR

Glucose and citric acid (with a molar ratio of 1:1.2) were esterified in a three‐necked flask for 3 h to form a crosslinked biomass prepolymer. Then, TA powder (15 wt.% based on the solid content of the biomass prepolymer) was mixed with the biomass prepolymer at room temperature and stirred vigorously for 30 min to obtain a tannic adhesive with a solid content of ≈45%. This adhesive was labeled as TAR.

### Preparation of the Reed‐Based Plastics

Rs fibers served as the reinforcement, whereas TAR served as the concrete. The mass ratio between TAR and the reed fibers was 1.5:8.5. First, Rs and P‐Rs were impregnated into TAR. Then, the excess liquid was drained, and the fibers were quickly dried at 60 °C to ensure that the concrete was fully distributed and wrapped around the reinforcement structure, thereby completing the initial filling of the reinforcement skeleton by the concrete. Subsequently, the dried TAR‐impregnated Rs and P‐Rs were directionally paved in a self‐made mold. An RCS‐inspired reed‐based plastic with dimensions of 100 mm (length) × 50 mm (width) × 3 mm (thickness) was prepared by pressing at room temperature under 30 MPa for 10 min, followed by hot‐pressing at 180 °C and 2.5 MPa for 7 min. The product was labeled as Si‐RP. Rs was laid on the surface as a protective layer, whereas P‐Rs was placed in its interior as a bonding layer. P‐Rs were separately prepared as a control sample with the same dimensions (100 mm length × 50 mm width × 3 mm thickness) and labeled as RP to compare the effects of the mechanisms according to the above method.

### Morphology and Structural Characterization

The microstructures of the Rs samples were observed using a scanning electron microscope (Sigma 300, ZEISS, Germany). The Derjaguin–Muller–Toporov (D–M–T) modulus map of the reed fibers was obtained using atomic force microscopy (AFM, Dimension Icon, Bruker, Germany). The cross‐sections of the reed‐based plastics were observed using a super‐depth 3D microscope (UDM, VHX‐7000, KEYENCE, Japan) to examine the distribution of TAR within the samples.

### Physical Characteristics

The surface wettability of the samples was measured using a surface tension meter (CA, OCA 20, Dataphysics, Germany). The cumulative pore volume, incremental pore volume, and porosity of the samples were measured using a high‐performance fully automatic mercury intrusion porosimeter (AutoPore IV 9510, Micromeritics, USA). The dimensional stability of the samples was measured by immersing them in water at room temperature and draining them until water no linger dripped from them. The thickness and weight of the samples were measured before and after WA, and the TS and WA were calculated using Equations (1) and (2), respectively.

(1) $$\mathrm{TS}=\frac{h_{2}-h_{1}}{h_{1}}\times 100\%$$

where *h_1_* and *h_2_* are the sample thicknesses before and after WA, respectively.

(2) $$\mathrm{WA}=\frac{m_{2}-m_{1}}{m_{1}}\times 100\%$$

where *m_1_* and *m_2_* represent the sample weights before and after WA, respectively.

### Chemical Characterization

The relative contents of cellulose, hemicellulose, and lignin in Rs were determined using a 912+ fiber analyzer and muffle furnace according to the standard washing method. The chemical composition and changes in the functional groups of the samples were characterized by Fourier‐transform infrared spectroscopy (FT‐IR, Nicolet iS20, Thermo Fisher Scientific, USA) over the wavenumber range of 400–4000 cm^−1^. The crystal structures of the reed‐based plastic samples were analyzed using 2D‐WAXD (HomeLab, Rigaku, Japan). The system was operated in transmission mode with a Cu‐Kα (λ = 1.54189 Å) microfocus source in a Pilatus 300 K detector under vacuum. The crystal structural changes of the samples were determined by monitoring the changes in the XRD peaks at 200°, 110°, 102°, and 1°–10° with a scanning rate of 5°/min and a 2θ range of 5°–60°. *φ* and *CI* were computed using Equations (3) and (4), respectively. Real‐time information on the quantity and type of functional groups present in the gases released during the combustion of the reed‐ and petroleum‐based plastics was obtained using a thermogravimetric analyzer and Fourier‐transform infrared spectrometer (TG‐IR, TGA55/Nicolet iS50, USA).

(3) $$\varphi =\frac{180^{\circ }-FWHM}{180^{\circ }}$$

where FWHM denotes the full width at half maximum of the azimuthal profiles obtained from the chosen equatorial reflections.

(4) $$CI=\frac{I_{crystalline}}{I_{crystalline}+I_{amorphous}}$$

where *I*_crystalline_ is the entire area of the crystalline cellulose and *I*_amorphous_ is the area of the amorphous cellulose.

### Mechanical Properties

The surface hardness of the samples was measured using a digital shore hardness tester. The mechanical properties of the reed‐based plastic samples in the dry and wet states were evaluated according to the Chinese National Standard (GB/T 17657‐2022 and GB/T 4897‐2015) using an electronic universal testing machine (Model 5982, INSTRON, USA). The tensile and bending test specimens were 90 mm long, 10 mm wide, and 3 mm thick, and the loading speed was 5 mm min^−1^.

### Molecular Dynamics (MD) Simulations

We used LAMMPS software combined with VMD, Packmol, and other open‐source auxiliary tools to complete the model construction, force field parameter assignment, energy minimization, and loading simulation and simulate and characterize the chemical and physical interactions between the reed fibers and TAR. A universal force field was used in LAMMPS. The system models included: 1) a TAR polymer structure composed of TA, glucose, and citric acid, 2) cellulose type Iβ crystals, which were constructed using the Cellulose‐builder tool, and SiO_2_ molecules with the corresponding mixing proportions on its surface, representing Rs, and 3) cellulose type II crystals constructed using the Cellulose‐builder tool, representing P‐Rs. These components were assembled into the RP and Si‐RP molecular structural systems via directional stacking. Packmol was used to optimize the spatial arrangement and eliminate significant atomic overlaps. The measured value of the simulation box was ≈28 × 28 × ZÅ^3^, with the Z‐direction automatically adjusted according to the layer structure. Prior to tensile loading, the system underwent sequential energy minimization (via the LAMMPS minimization command) and thermodynamic equilibration. NVT ensemble equilibration preceded NPT equilibration under isotropic pressure control to replicate the experimental conditions. Periodic boundary conditions were applied along the x‐ and z‐axes, whereas the y‐direction boundaries remained nonperiodic (free) to enable structural deformation analysis.

## Conflict of Interest

The authors declare no conflict of interest.

## Author Contributions

Y.H. contributed to data curation, formal analysis, and wrote the original draft. H.L. performed investigation and formal analysis. M.L. contributed to software and formal analysis. K.L. performed formal analysis. Y.Z. performed the investigation. Z.Z. performed supervision, project administration, and administered funding acquisition, resources, and wrote‐reviewed and edited the manuscript. Y.Z. performed conceptualization, methodology, and funding acquisition. Y.W. contributed to funding acquisition, project administration, and wrote‐reviewed, and edited the final manuscript.

## Supporting information



Supporting Information

## Data Availability

The data that support the findings of this study are available from the corresponding author upon reasonable request.
