# Supplementary material for: Inspired by Reinforced‐Concrete Structures: High‐Strength, Stable, Sustainable Reed‐Based Plastics
Source: Adv Sci (Weinh). 2025 Aug 11;12(40):e11564. doi: 10.1002/advs.202511564 (PMC12561195; doi:10.1002/advs.202511564)
Supplement: Supplementary file 1 — Supporting Information [file ADVS-12-e11564-s001.docx]

**Supplementary Information**

**Inspired by reinforce-concrete structure: a high-strength, stable, sustainable reed-based plastics**

Yuhui Huang^1^, Handong Li^2^, Meng Li^1^, Kui Li^1^, Yawen Zhang^3^, Zhen Zhang^1*^, Yingfeng Zuo^1*^, Yiqiang Wu^1*^

1. State Key Laboratory of Utilization of Woody Oil Resource, Central South University of Forestry and Technology, Changsha, Hunan 410004, P.R. China

2. Huidong County Forestry Technology Promotion Station, Huizhou, Guangdong 516300, P.R. China

3. College of Furniture and Art Design, Central South University of Forestry and Technology, Changsha, Hunan 410004, P.R. China

*Corresponding author: [zhangzhen_0428@163.com](mailto:zhangzhen_0428@163.com) (Zhen Zhang), [zuoyf1986@163.com](mailto:zuoyf1986@163.com) (Yingfeng Zuo), [wuyq0506@126.com](mailto:wuyq0506@126.com) (Yiqiang Wu)


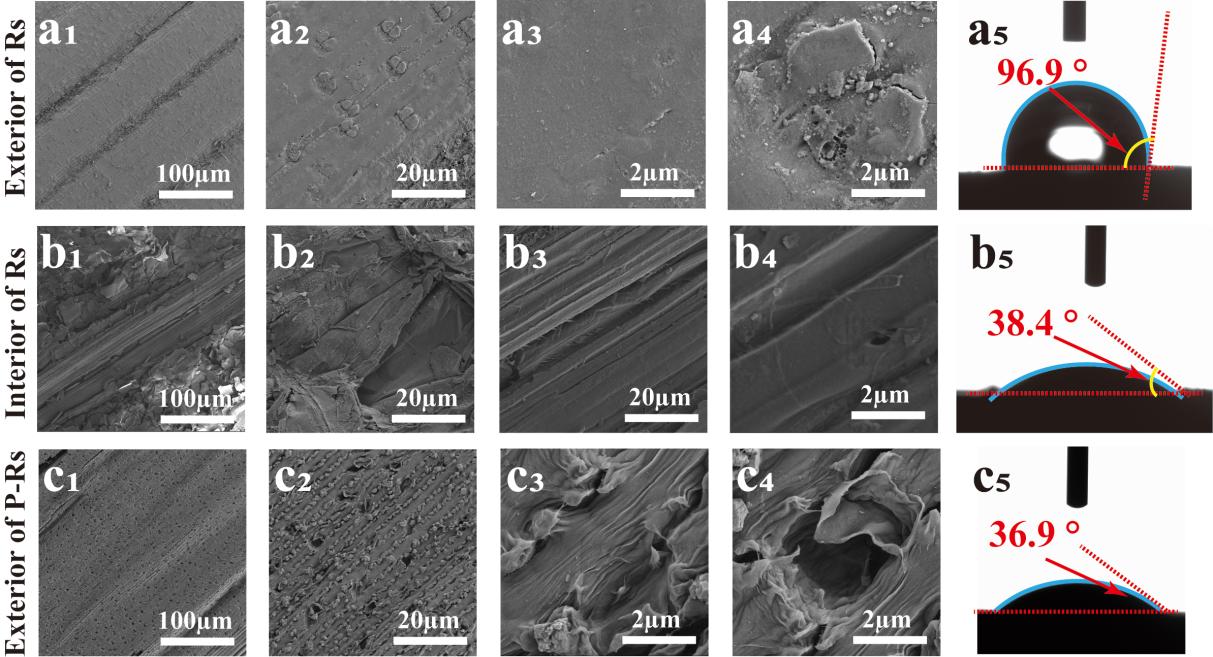


**Supplementary Fig. S1.** a) The SEM image and contact angle of the outer surface of Rs. b) The SEM image and contact angle of the inner surface of Rs. c) The SEM image and contact angle of the outer surface of P-Rs.


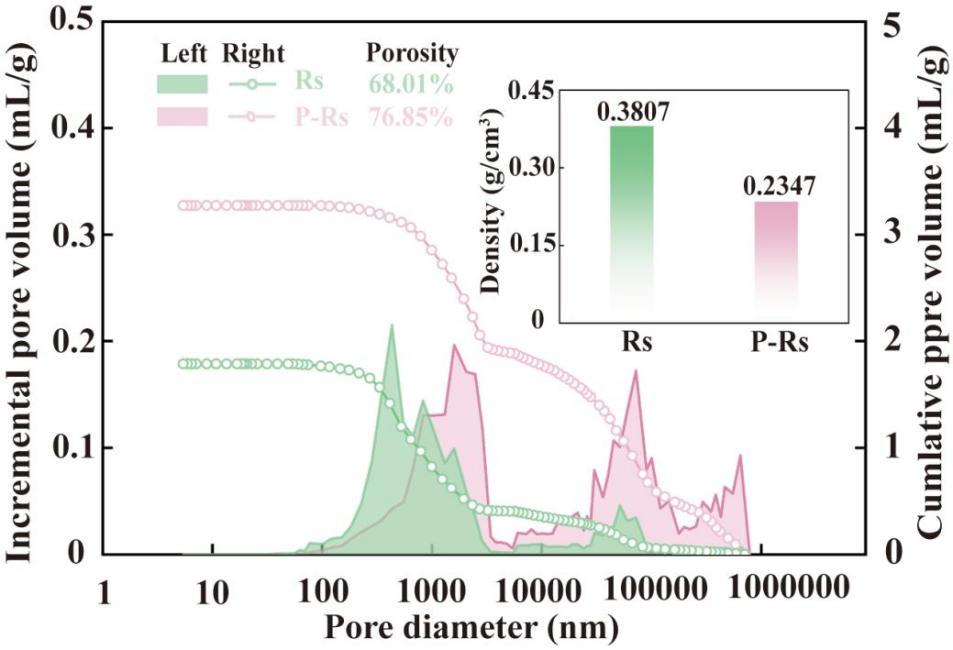


**Supplementary Fig. S2.** High-performance fully automatic mercury intrusion porosimeter characterization of Rs and P-Rs, including incremental pore volume, cumulative pore volume, porosity, and density.


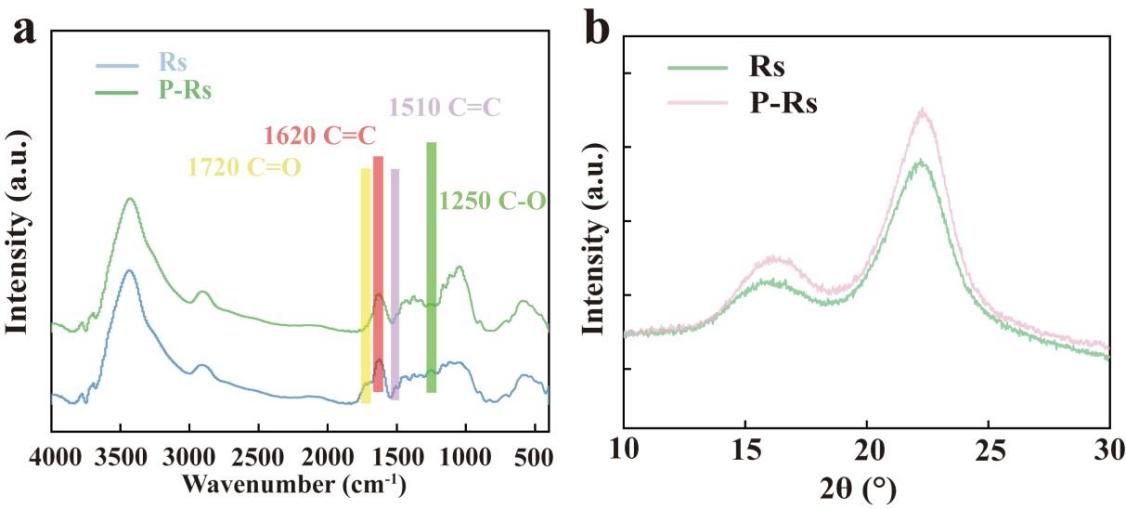


**Supplementary Fig. S3.** a) FT-IR spectra of Rs and P-Rs. b) Crystallinity characterization of Rs and P-Rs.


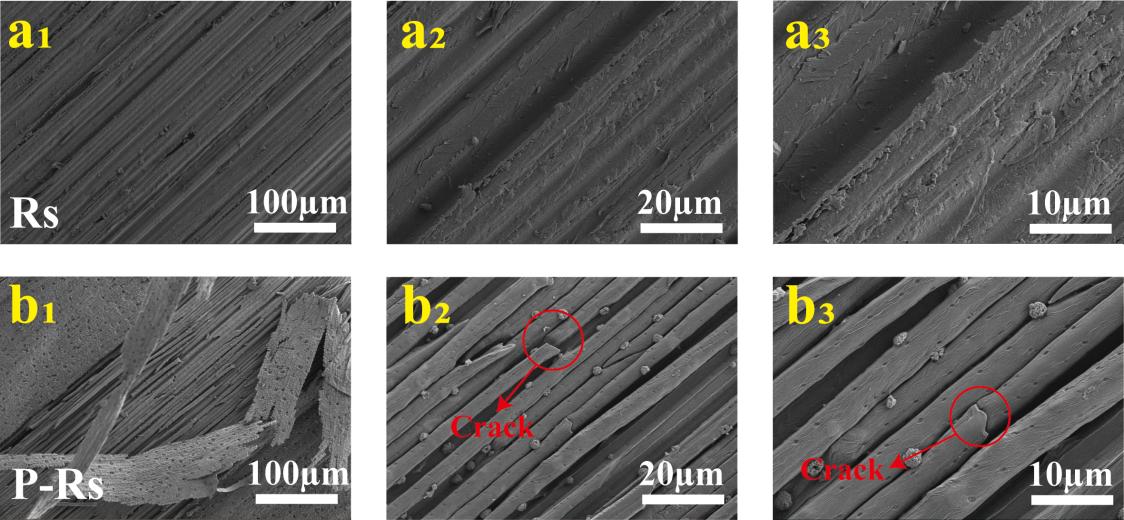


**Supplementary Fig. S4.** Changes of reed fiber before and after treatment. a) Natural reed fiber. b) Reed fiber after alkali treatment.


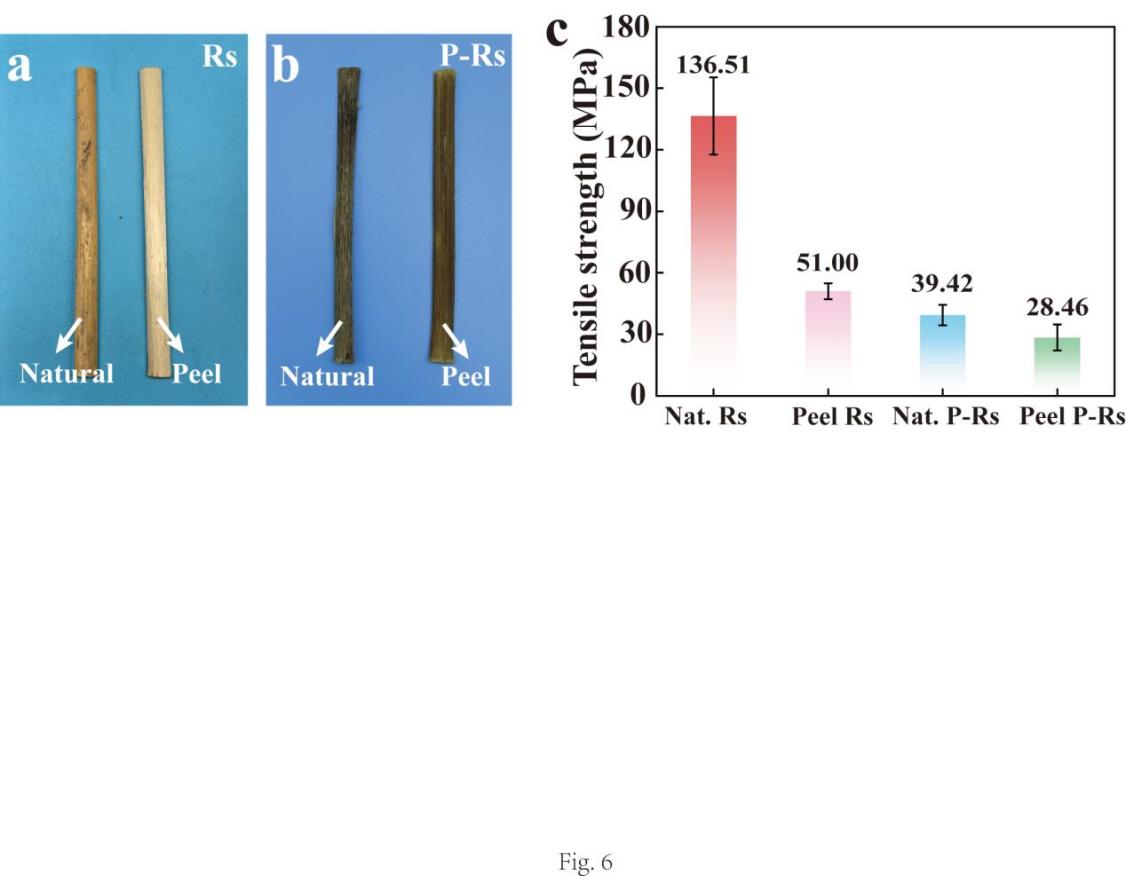


**Supplementary Fig. S5.** a) Physical drawings of Rs and peeled Rs. b) Physical drawings of P-Rs and peeled P-Rs after pretreatment. c) Tensile strength of Rs and P-Rs before and after peeling.


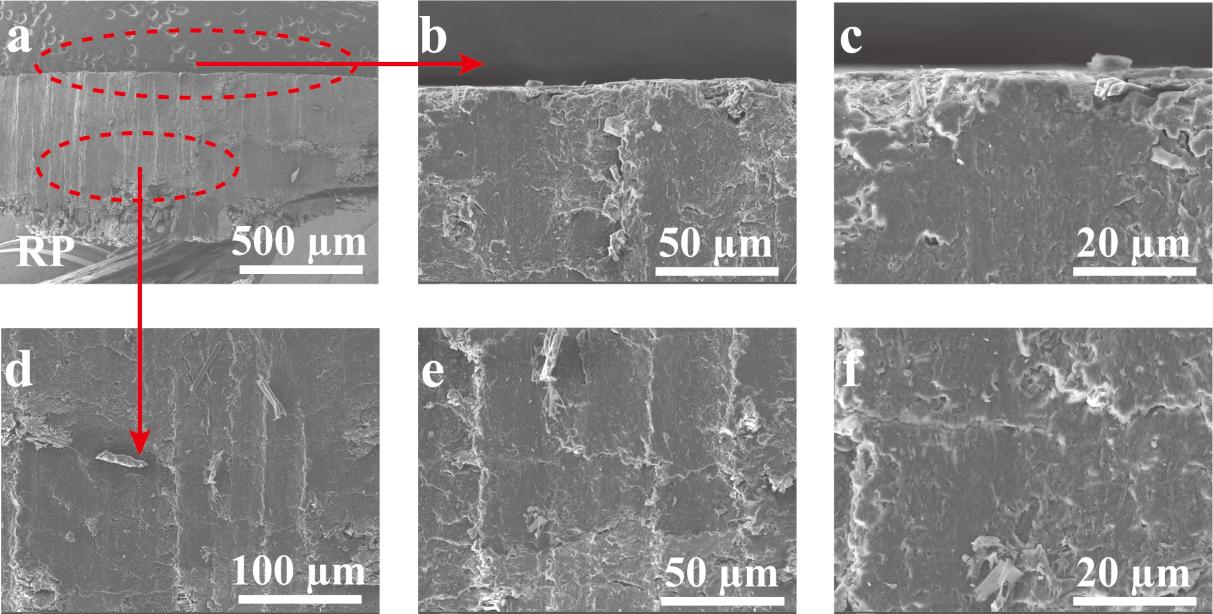


**Supplementary Fig. S6.** The SEM images of RP cross section at different magnification.


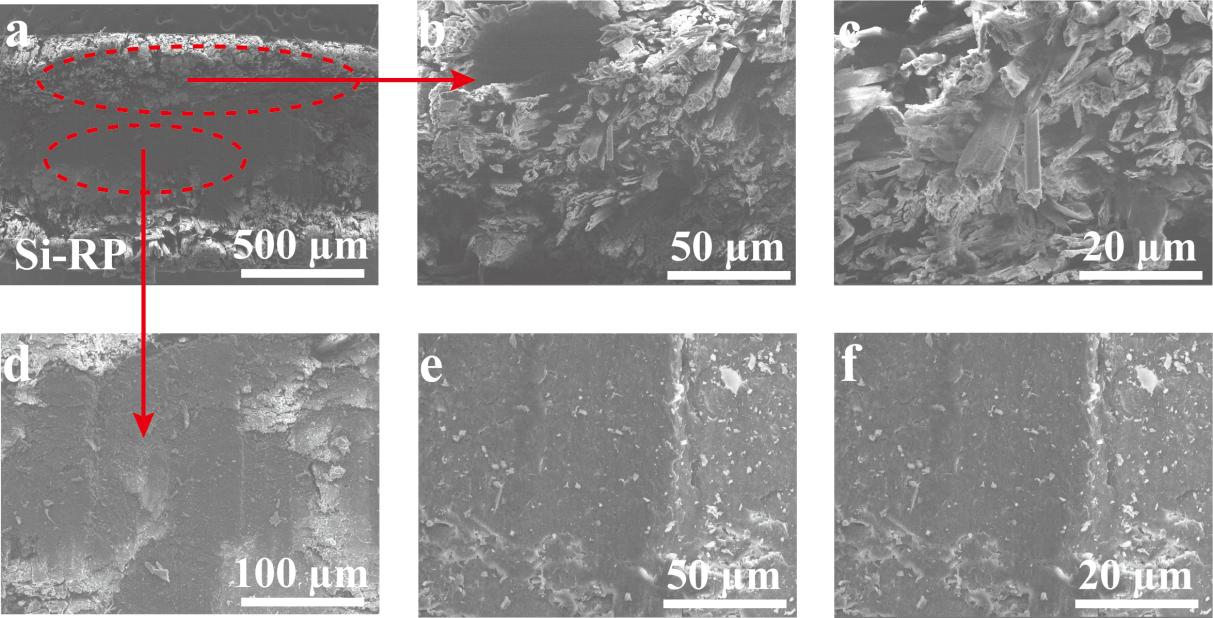


**Supplementary Fig. S7.** The SEM images of Si-RP cross section at different magnification.


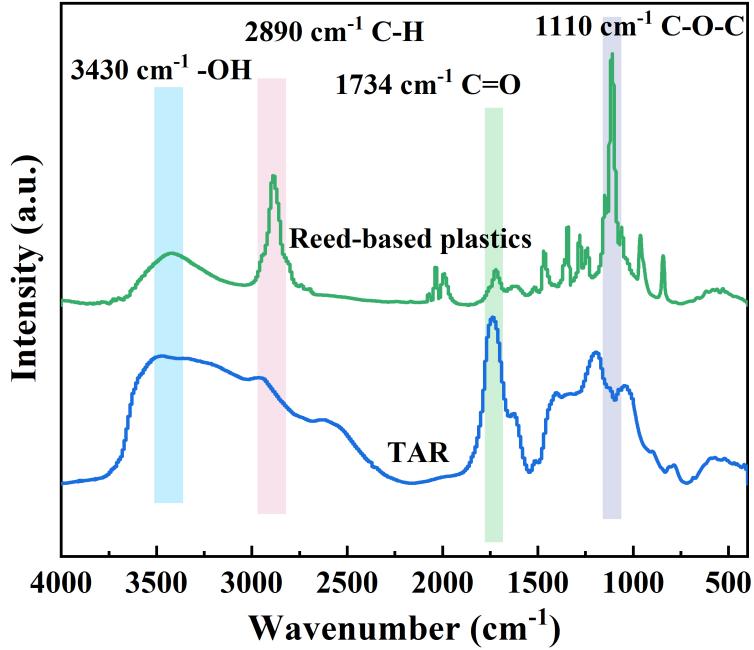


**Supplementary Fig. S8.** FT-IR spectra of TAR and reed-based plastics.


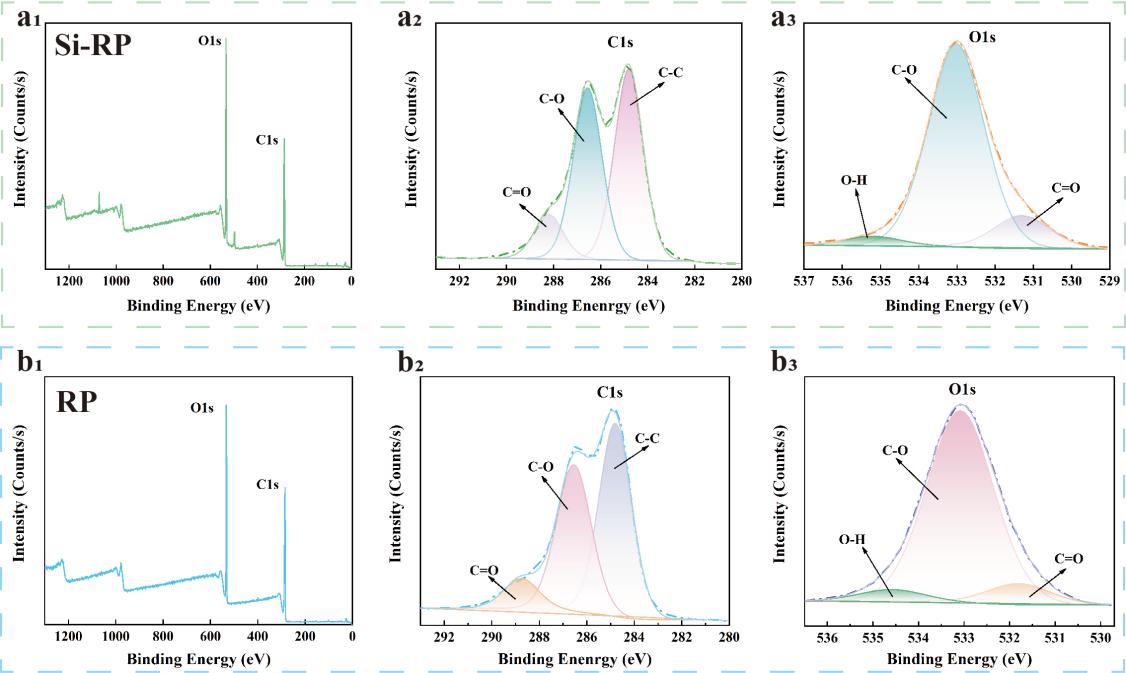


**Supplementary Fig. S9.** XPS characterization of Si-RP and RP.


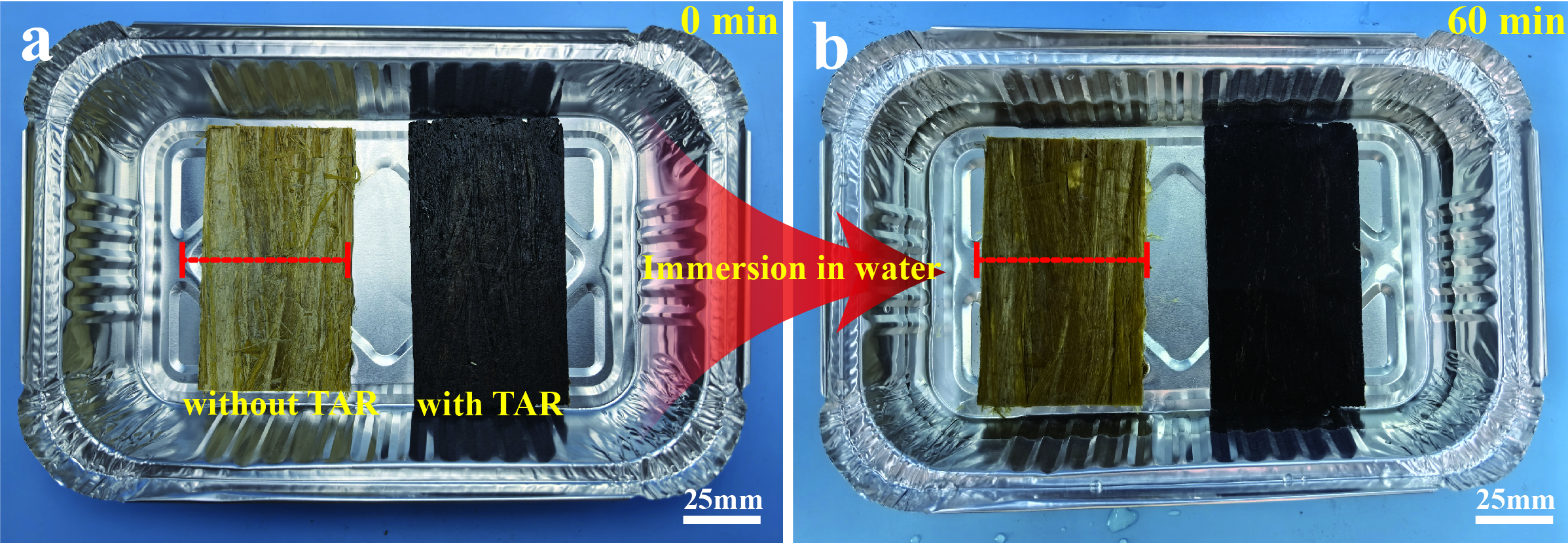


**Supplementary Fig. S10.** Images of the compact TAR-free material directly hot-pressed from P-Rs and the RP before (a) and after (b) immersion in water for 60 min.


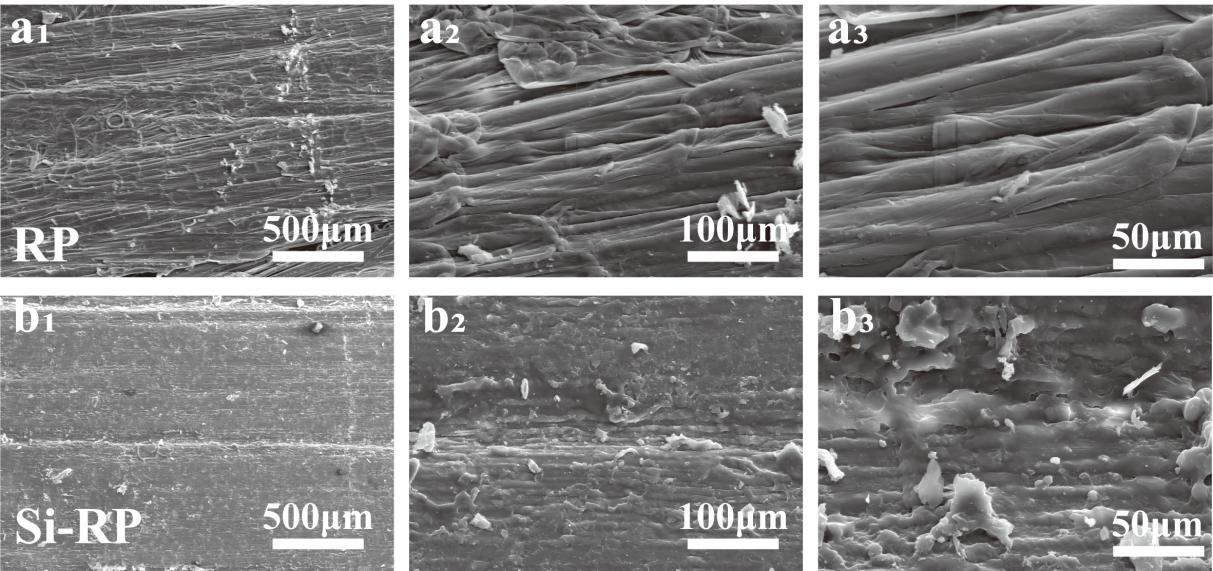


**Supplementary Fig. S11.** The SEM images of RP (a) and Si-RP (b) surfaces at different magnification.


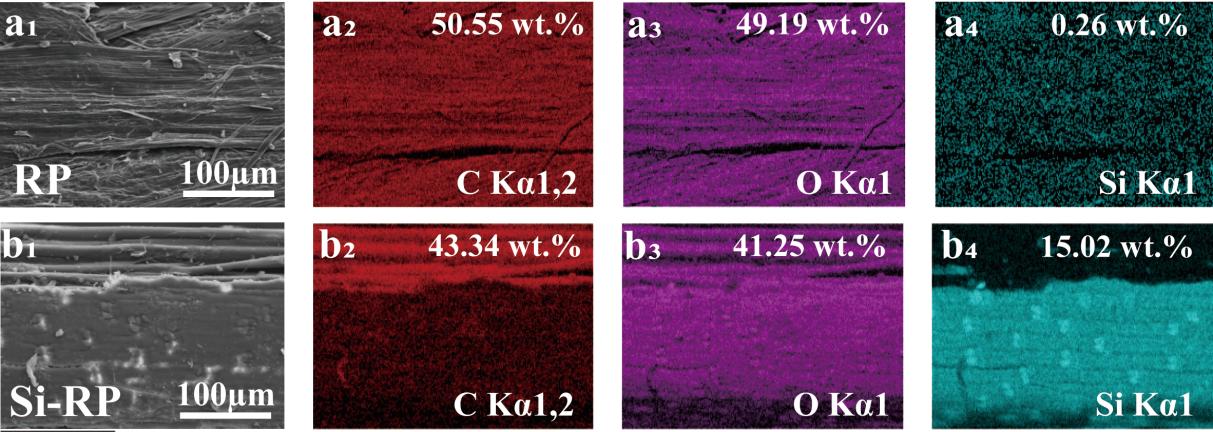


**Supplementary Fig. S12.** Distribution diagrams of C, O, and Si elements in the outer of RP (a) and Si-RP (b).


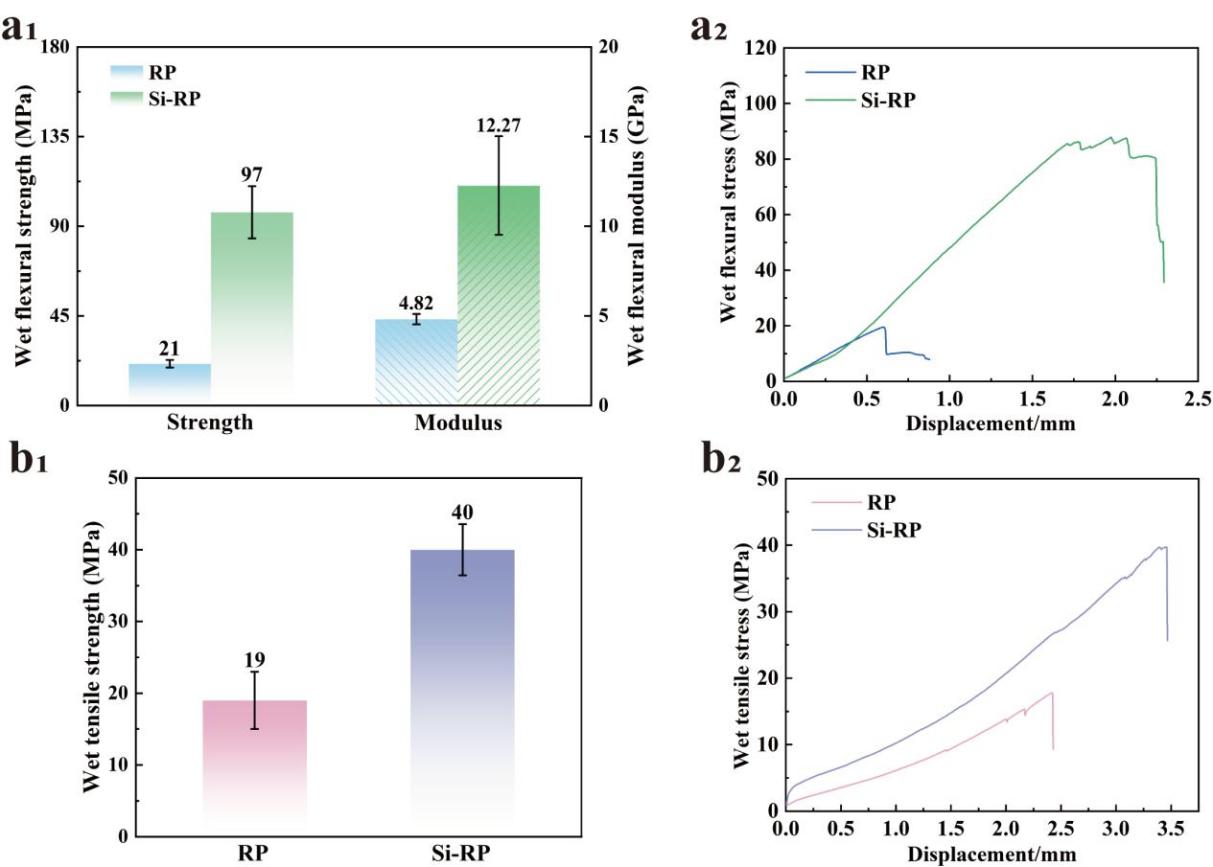


**Supplementary Fig. S13.** Mechanical properties of RP and Si-RP after 24h water immersion at room temperature, including wet flexural strength and modulus (a_1_), wet flexural stress displacement curve (a_2_), wet tensile strength (b_1_) and wet tensile stress displacement curve.


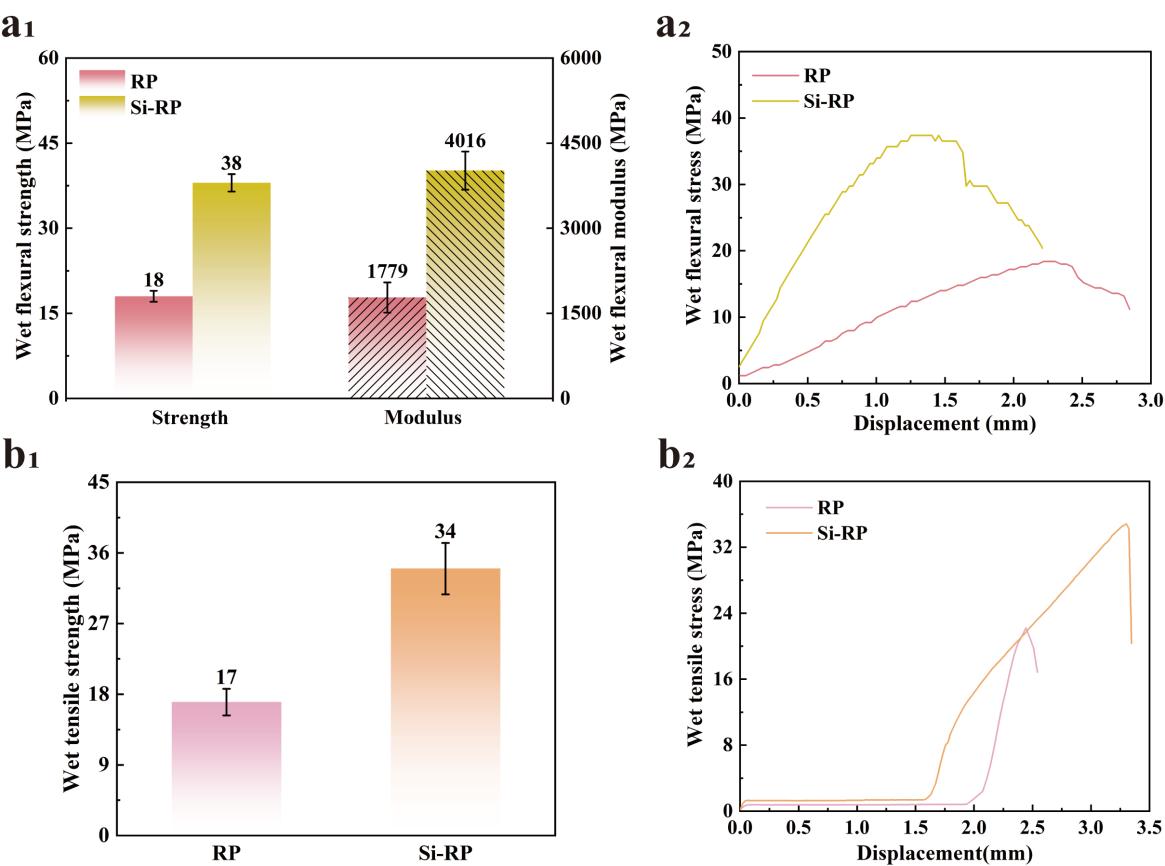


**Supplementary Fig. S14.** Mechanical properties of RP and Si-RP after being immersed in water for 2h at 70℃, including wet flexural strength and modulus (a_1_), wet flexural stress displacement curve (a_2_), wet tensile strength (b_1_) and wet tensile stress displacement curve.


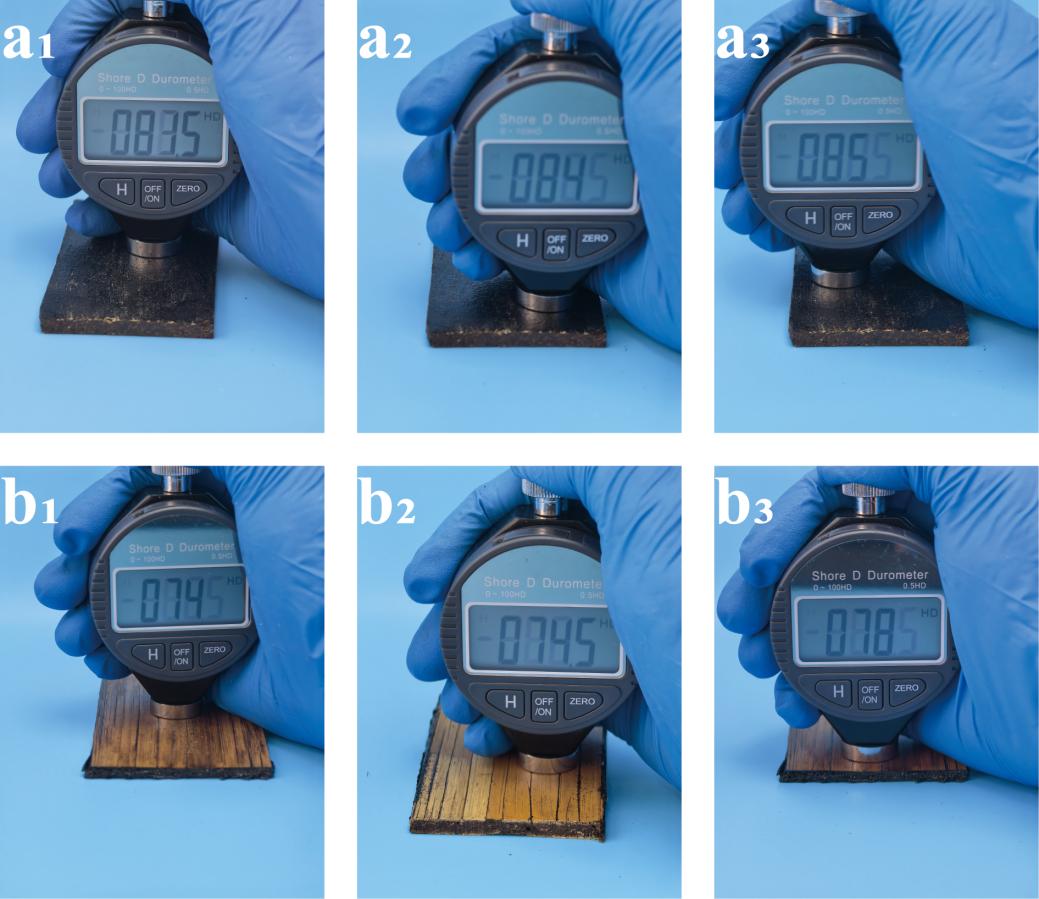


**Supplementary Fig. S15.** Shore D hardness of RP (a) and Si-RP (b).


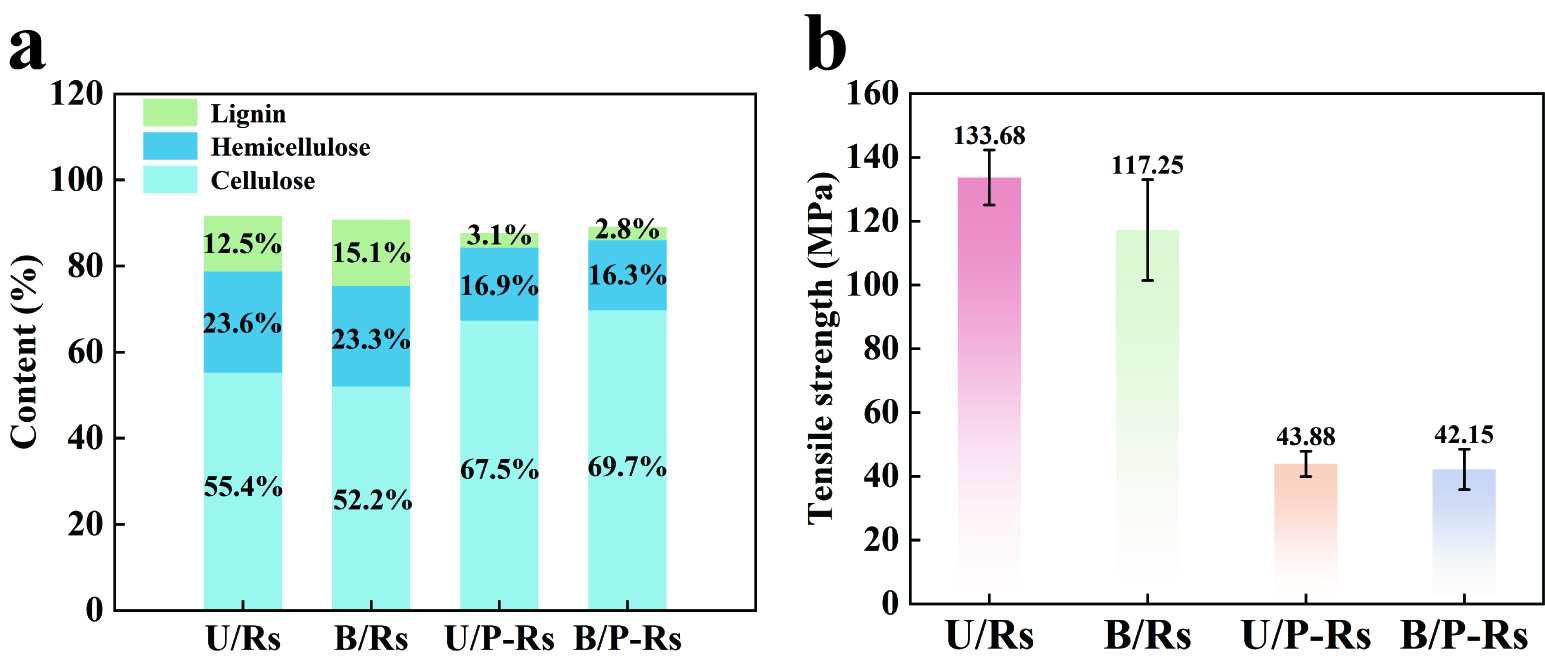


**Supplementary Fig. S16.** The chemical components contents and tensile strength of different parts of the reed straw, including the changes in cellulose, hemicellulose, and lignin contents of the upper straw (U/Rs) and basal straw (B/Rs) of reed before and after alkaline treatment (a), the tensile strength changes of the U/Rs and B/Rs of reed before and after alkaline treatment (b).


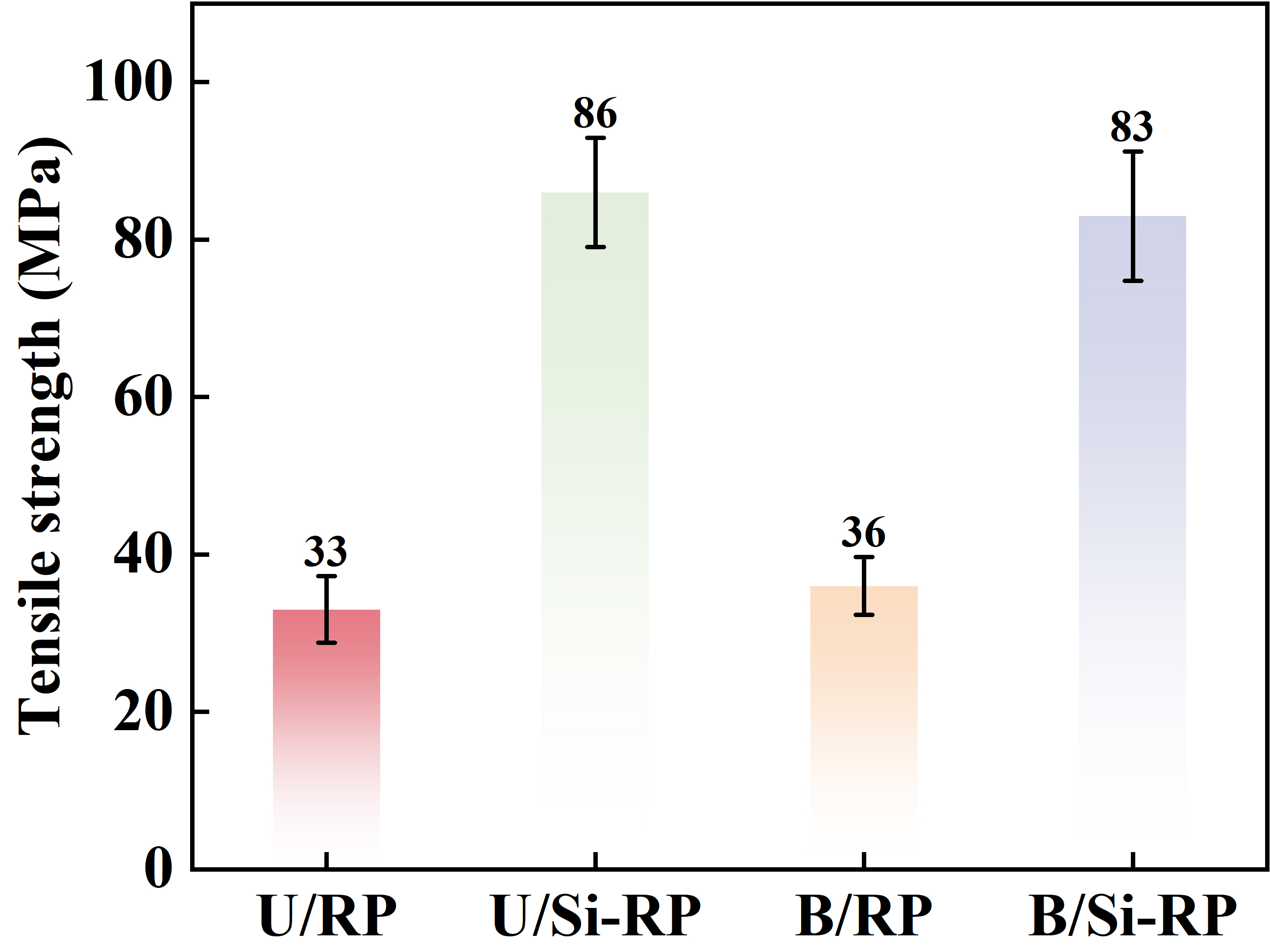


**Supplementary Fig. S17.** RP and Si-RP prepared from different parts of the reed straw, including RP systems fabricated solely from the upper or lower stem, and Si-RP systems fabricated solely from the upper or lower stem.


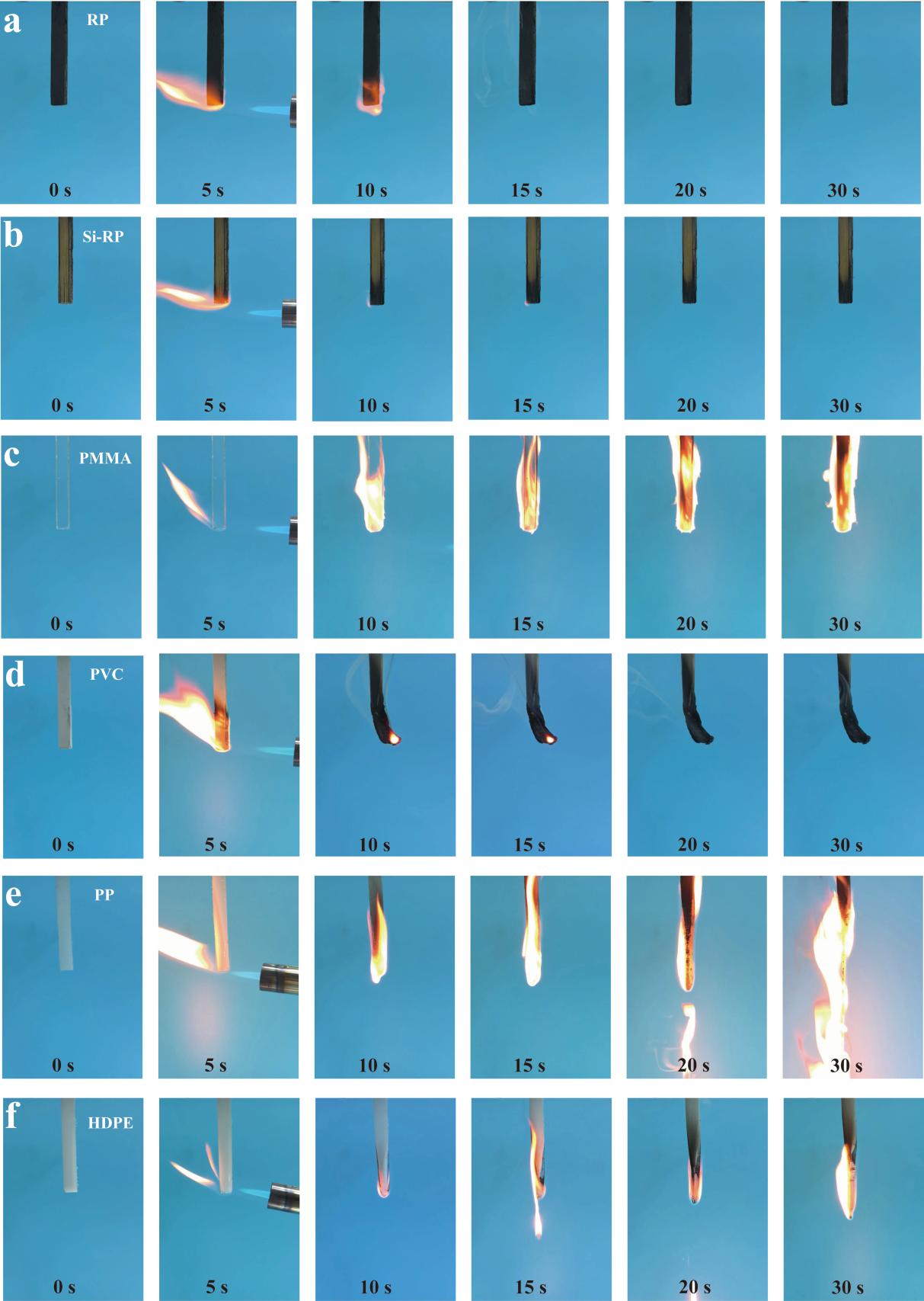


**Supplementary Fig. S18.** Comparison of the flame-retardant properties between RP (a), Si-RP (b), PMMA (c), PVC (d), PP (e), and HDPE (f) during ignition and combustion.


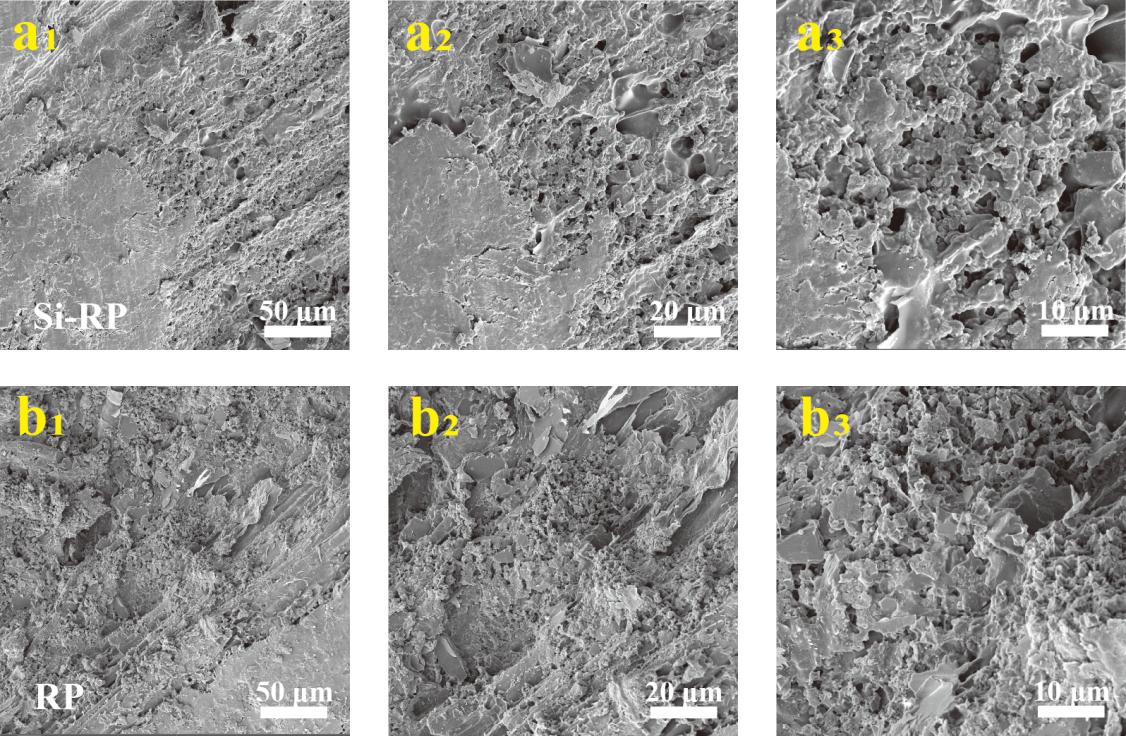


**Supplementary Fig. S19.** SEM micrographs of char for Si-RP (a) and RP (b) after burning test.


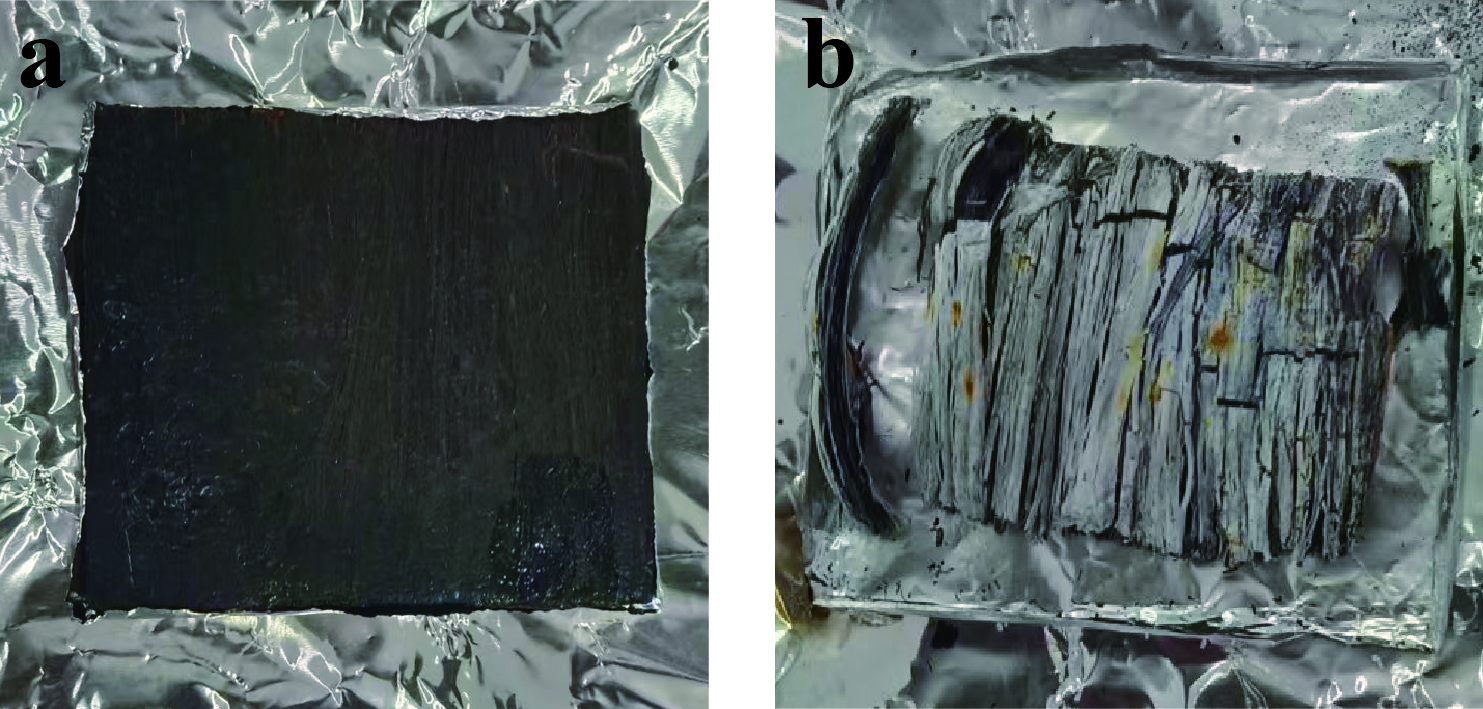


**Supplementary Fig. S20.** Images of char for reed-based plastic after cone calorimeter test.

**Supplementary Table S1.**  Elemental composition of C1 s and O1 s in reed-based plastics.

| Reed-based plastics | Elemental composition | | Peak BE | Atomic% |
| --- | --- | --- | --- | --- |
| Si-RP | C1 s | C-C | 284.80 | 47.08 |
|  |  | C-O | 286.55 | 42.14 |
|  |  | C=O | 288.21 | 10.78 |
|  | O1 s | O-H | 535.22 | 3.78 |
|  |  | C-O | 533.02 | 82.77 |
|  |  | C=O | 531.30 | 13.45 |
| RP | C1 s | C-C | 284.80 | 50.50 |
|  |  | C-O | 286.54 | 41.06 |
|  |  | C=O | 288.75 | 8.44 |
|  | O1 s | O-H | 534.59 | 5.29 |
|  |  | C-O | 533.08 | 85.77 |
|  |  | C=O | 531.80 | 8.94 |

**Supplementary Table S2.** Comparison of flexural and tensile properties of RP, Si-RP and the compact TAR-free materials.

| Composites | Flexural Strength  (MPa) | Flexural  Modulus  (GPa) | Tensile  Strength  (MPa) | Density  (g/cm^3^) |
| --- | --- | --- | --- | --- |
| RP | 76 ±2.97 | 10.11 ± 0.61 | 35 ± 5.59 | 1.08 |
| Si-RP | 116 ± 8.05 | 13.54 ± 0.67 | 81 ± 9.85 | 0.85 |
| Compact TAR-free material | 12 ± 3.72 | 17.34 ± 0.81 | 24 ± 4.76 | 1.05 |

**Supplementary Table S3.** Comparison of the mechanical properties of reed-based plastic with other natural fiber composites.^[1-9]^

| Composites | Flexural Strength (MPa) | Flexural Modulus (GPa) | Tensile Strength (MPa) | Density  (g/cm^3^) |
| --- | --- | --- | --- | --- |
| Si-RP | 116 | 13.54 | 81 | 0.85 |
| RP | 76 | 10.11 | 35 | 1.08 |
| Bamboo/Wood-Chitosan/Tannin adhesive | 100 | 10.00 | - | - |
| Bamboo Particle-Epoxidized Soybean Oil Adhesive | 20 | 2.86 | - | 0.79 |
| Bamboo Fiber-Lignin | 97 | 261 | 41 | 0.81 |
| Bamboo Fiber Bundle-Self Adhesion | 101 | 13.30 | 45 | 1.42 |
| Bamboo Fiber-Poly(Lactic Acid) | 80 | 6.34 | 55 | 1.28 |
| Bamboo Fiber Bundle-PF Resin | 519 | 38.90 | 853 | 1.35 |
| Flax Fiber-Chitosan Films | 90 | 3.27 | 94 | 1.54 |
| Hemp Fiber-Epoxidized Soybean Oil | 116 | 7.89 | 84 | 1.25 |
| Ramie Fiber-Poly(Lactic Acid) | 97 | 8.12 | 70 | 1.25 |

**Supplementary Table S4.** Comparison of specific strength of reed-based plastic with other natural fiber composites.^[2-9]^

| Composites | Specific  Flexural Strength (MPa/g·cm^-3^) | Specific  Flexural Modulus (GPa/g·cm^-3^) | Specific  Tensile Strength (MPa/g·cm^-3^) |
| --- | --- | --- | --- |
| Si-RP | 136 | 15 | 95 |
| RP | 70 | 9 | 32 |
| Bamboo Particle-Epoxidized Soybean Oil Adhesive | 25 | 4 | - |
| Bamboo Fiber-Lignin | 120 | 3 | 51 |
| Bamboo Fiber Bundle-Self Adhesion | 71 | 9 | 31 |
| Bamboo Fiber-Poly(Lactic Acid) | 63 | 5 | 43 |
| Bamboo Fiber Bundle-PF Resin | 384 | 28 | 632 |
| Flax Fiber-Chitosan Films | 58 | 2 | 61 |
| Hemp Fiber-Epoxidized Soybean Oil | 92.8 | 6 | 67 |
| Ramie Fiber-Poly(Lactic Acid) | 77.6 | 6 | 56 |

**Supplementary Table S5.** Cone calorimetry data of reed-based plastic.

| Samples | TTI  (s) | pHRR  (kW/m^2^) | THR  (MJ/m^2^) | TSR  (m^2^/m^2^) | TSP  (m^2^) |
| --- | --- | --- | --- | --- | --- |
| Reed-based plastic | 37 | 395 | 20 | 63 | 0.24 |
| PMMA | 28 | 889 | 45 | 333 | 1.26 |
| PVC | 35 | 150 | 26 | 1420 | 5.40 |
| PP | 21 | 1098 | 64 | 1044 | 3.97 |
| HDPE | 31 | 758 | 74 | 996 | 3.78 |

**Supplementary Table S6.** Coparison of thermal stability and flame retardancy between reed-based plastic and other natural fiber composites.^[10-13]^

| Composites | Maximum Rate of Thermal Decomposition  (%/min) | TSP  (m^2^) | THR  (MJ/m^2^) |
| --- | --- | --- | --- |
| Reed-based Plastic | 332 | 0.24 | 20 |
| Bamboo Fiber-PF Resin-Tannic Acid+Fe^2+^ | - | 2.7 | 181 |
| Ramie Fiber-Epoxy-Phytic Acid+Boric Acid+Xylitol | 283 | 12.1 | 47 |
| Wood Fiber-UF Resin-THE-TT+Phosphoric Acid | 350 | 3.0 | 9 |
| Bamboo Fiber-PLA-Triazine Derivative+Ammonium Polyphosphate | 373 | - | 53 |

# References

1. S. Jiang, S. Liu, G. Du, S. Wang, X. Zhou, J. Yang, Z. Shi, Z. Yang, T. Li, Chitosan-tannin adhesive: Fully biomass, synthesis-free and high performance for bamboo-based composite bonding, *Int. J. Biol. Macromol.* **2023**, 230, 123115.
2. L. Zhang, Y. Chen, S. Geng, Z. Huang, R. Qiu, T. Chen, W. Liu, Development of plant oil-based adhesives for formaldehyde-free bamboo particleboards, *Ind. Crop. Prod.* **2024**, 210, 118146.
3. Y. Ren, Y. Zhong, Y. Yang, H. Huo, L. Zhang, J. Zhang, K. Huang, Z. Zhang, Green recyclable biocomposite prepared from lignin and bamboo, *J. Clean. Prod.* **2024**, 449, 141710.
4. S. Ochi, Mechanical properties of bamboo fiber bundle-reinforced bamboo powder composite materials, *Eur. J. Wood Wood Prod.* **2022**, 80, 263-275.
5. Y. Yang, H.Wan, B. Wang, B. Wang, K. Chen, H. Tan, C. Sun, Y. Zhang, Preparation and properties of bamboo fiber/polylactic acid composite modified with polycarbodiimide, *Ind. Crop. Prod.* **2024**.218, 118829.
6. J. Hu, J. Wu, Y. Huang, Y. He, J. Lin, Y. Zhang, Y. Zhang, Y. Yu, W. Yu, Super-strong biomimetic bulk bamboo-based composites by a neural network interfacial design strategy, *Chem. Eng. J.* **2023**, 475, 146435.
7. A. Rath, B. Grisin, T.D. Pallicity, L. Glaser, J. Guhathakurta, N. Oehlsen, S. Simon, S. Carosella, P. Middendor, L. Stegbauer, Fabrication of chitosan-flax composites with differing molecular weights and its effect on mechanical properties, *Compos. Sci. Technol.* **2023**, 235, 109952.
8. W. Liu, M. Fei, Y. Ban, A. Jia, R. Qiu, J. Qiu, Concurrent improvements in crosslinking degree and interfacial adhesion of hemp fibers reinforced acrylated epoxidized soybean oil composites, *Compos. Sci. Technol.* **2018**. 160, 60-68.
9. J. Zhan, G. Wang, J. Li, Y. Guan, G. Zhao, H. Naceur, D. Coutellier, J. Lin, Effect of the compatilizer and chemical treatments on the performance of poly(lactic acid)/ramie fiber composites, *Compos. Commun*. **2021**, 27, 100843.
10. X. Li, W. Lei, F. Rao, Y. Bao, S. Liao, Y. Zhang, N. Li, Y. Zhang, Y. Chen, A green-modification strategy for high-performance bamboo-based composites via persimmon lacquer and iron ion complexation, *Nano Lett.* **2025**, 25, 3414-3421.
11. J. Yang, Y. Sun, W. Song, Y. Liu, A novel phosphorus/boron-containing flame retardant for improving the flame retardancy of ramie fiber reinforced epoxy composites, *Constr. Build. Mater.* **2024**, 451, 138814.
12. D. Yan, D. Chen, J. Tan, L. Yuan, Z. Huang, D. Zou, P. Sun, Q. Tao, J. Deng, Y. Hu, Synergistic flame retardant effect of a new N-P flame retardant on poplar wood density board, *Polym. Degrad. Stabil.* **2023**, 211, 110331.
13. Q. Niu, X.Yue, W.Cao, Z. Guo, Z. Fang, P. Chen, J. Li, Interfacial silicon‑nitrogen aerogel raise flame retardancy of bamboo fiber reinforced polylactic acid composites, *Int. J. Biol. Macromol.* **2022**, 222, 2697-2708.
